# Photocatalytic regioselective C–H bond functionalizations in arenes

**DOI:** 10.1039/d4sc07491b

**Published:** 2024-12-09

**Authors:** Jun Hu, Suman Pradhan, Satyadeep Waiba, Shoubhik Das

**Affiliations:** a Department of Chemistry, University of Bayreuth Universitätsstraße 30 95447 Bayreuth Germany shoubhik.das@uni-bayreuth.de

## Abstract

The direct functionalization of C–H bonds has revolutionized the field of synthetic organic chemistry by enabling efficient and atom-economical modification of arenes by avoiding prefunctionalization. However, the inherent challenges of inertness and regioselectivity in different C–H bonds, particularly for distal positions, necessitate innovative approaches. In this aspect, photoredox catalysis by utilizing both transition metal and organic photocatalysts has emerged as a powerful tool for addressing these challenges under mild reaction conditions. This review provides a comprehensive overview of recent progress in regioselective C–H functionalization in arenes *via* photocatalysis. Emphasizing the strategies for achieving *ortho-*, *meta-*, and *para*-selectivity, we explore the mechanistic insights, catalyst designs, and the novel methodologies that have expanded the scope of C–H bond functionalization. This discussion aims to offer valuable perspectives for advancing the field and developing more efficient and sustainable synthetic methodologies.

## Abstract 2

The direct functionalization of C–H bonds has revolutionized the field of synthetic organic chemistry by enabling efficient and atom-economical modification of arenes by avoiding prefunctionalization.

## Graphical abstract

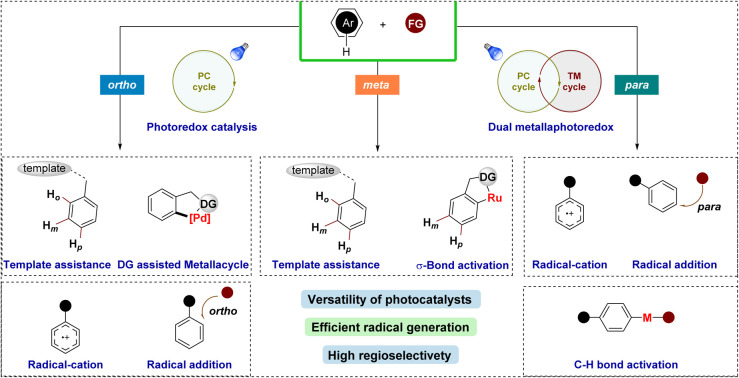

## 1. Introduction

The direct functionalization of C–H bonds in arenes is a pivotal technique in contemporary organic synthesis, presenting an attractive and efficient strategy for incorporating diverse functionalities into arenes.^1–7^ These approaches generate derivatives with potentially enhanced biological activity, making them highly valuable in the fields of drug discovery and biochemistry. Additionally, direct C–H bond functionalization strategies offer an opportunity to increase molecular complexity using readily available starting materials, which obviates the necessity for intricate prefunctionalization steps.^8–11^ This approach is particularly advantageous since it minimizes the environmental and economic impact of organic synthesis and promotes the principles of green chemistry. In this respect, it is worth mentioning that direct C–H functionalization offers substantial advantages; however, it is tempered by two fundamental challenges: selective activation of the C–H bond and attaining high regioselectivity.^12^ The inert nature of most of the C–H bonds makes them poorly reactive, which often requires stringent reaction conditions such as elevated temperature and the use of strong oxidants. At the same time, the prevalence of C–H bonds in organic molecules and the subtle reactivity differences among multiple C–H bonds present significant challenges to achieving high regioselectivity in direct C–H bond functionalization. In this aspect, transition metal catalysis provides a highly desirable approach for proximal C–H bond activation through the formation of conformationally rigid five- or six-membered metallacycle intermediates, which are both kinetically and thermodynamically favored.^13,14^ However, this strategy is less applicable for selectively targeting C–H bonds that are distant from existing functional groups, since it requires the formation of much larger and less feasible metallacycles. Nevertheless, various research groups have developed methods to achieve regioselective functionalization at remote *meta*- and *para*-positions of benzene rings. These techniques often employ directing groups or exploit weak interactions to guide the functionalization process.^15–17^ Recently, there have been reports of undirected C–H bond functionalization at remote sites where the choice of reagents, ligands and catalysts plays a crucial role in determining the regioselectivity.^18^

**Fig. 1:**
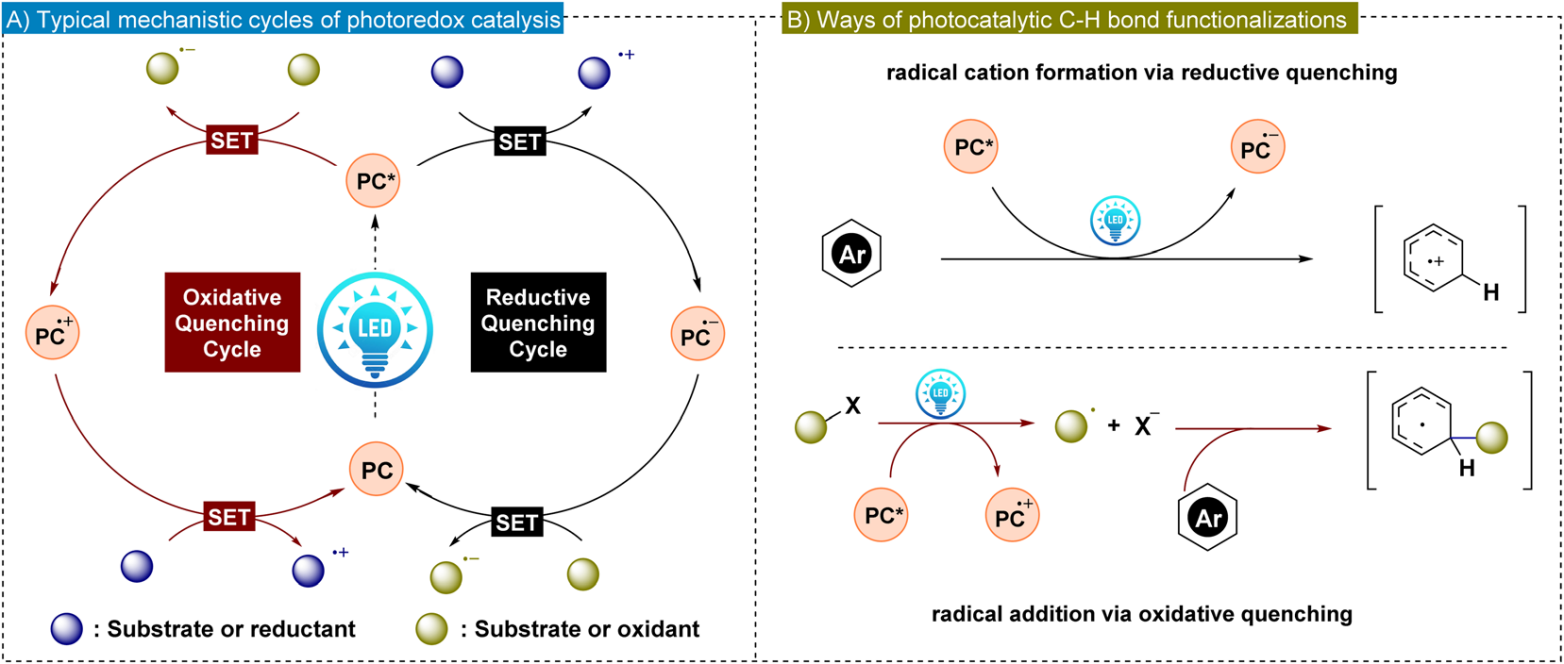
(A) General mechanism of photoredox catalysis. (B) Photoredox catalysis mediated arene C–H bond activation strategies.

Although transition metal-catalyzed C–H bond functionalization has effectively addressed the issue of regioselectivity, there remains a strong demand for achieving highly regioselective C–H functionalization reactions under mild reaction conditions.^19^ In this regard, visible light-promoted photoredox catalysis has emerged as a promising solution due to its environmentally benign nature and efficiency in chemical transformations.^20–22^ In general, photo-induced organic reactions are catalyzed by either transition metal complexes or organic photocatalysts.^23,24^ Among transition metal photocatalysts, iridium and ruthenium complexes are extensively studied. These metal complexes benefit from the lower energy of the ligand's π* orbital, facilitating the metal–ligand charge transfer process.^25^ Alternatively, metal-free organic photocatalysts are gaining significant attention due to their low price and easy availability.^26–30^ Notable organic photocatalysts include benzophenones, acridinium salts, rhodamines and cyanoarenes. Both types of photocatalysts (PCs) harness sunlight as an inexpensive and renewable energy source. Upon irradiation of light, PCs form long-lived photoexcited states (PC*), which exhibit increased reactivity in electron-transfer events compared to their ground states. In reductive quenching, PC* accepts an electron from a substrate or reductant, forming a radical anion, which subsequently undergoes oxidation. Conversely, oxidative quenching is commonly involved in C–H bond functionalization *via* photoredox reactions. Here, PC* donates an electron to a substrate or an oxidant, generating the radical cation that is later reduced (Fig. 1A). These single electron transfer (SET) processes enhance radical generation, significantly advancing the radical-based transformations in synthetic organic chemistry (Fig. 1B). In addition, the tunability of light as an energy source in photoredox catalysis provides precise control over reaction conditions, enabling enhanced selectivity for functionalization in aromatic systems.

**Fig. 2:**
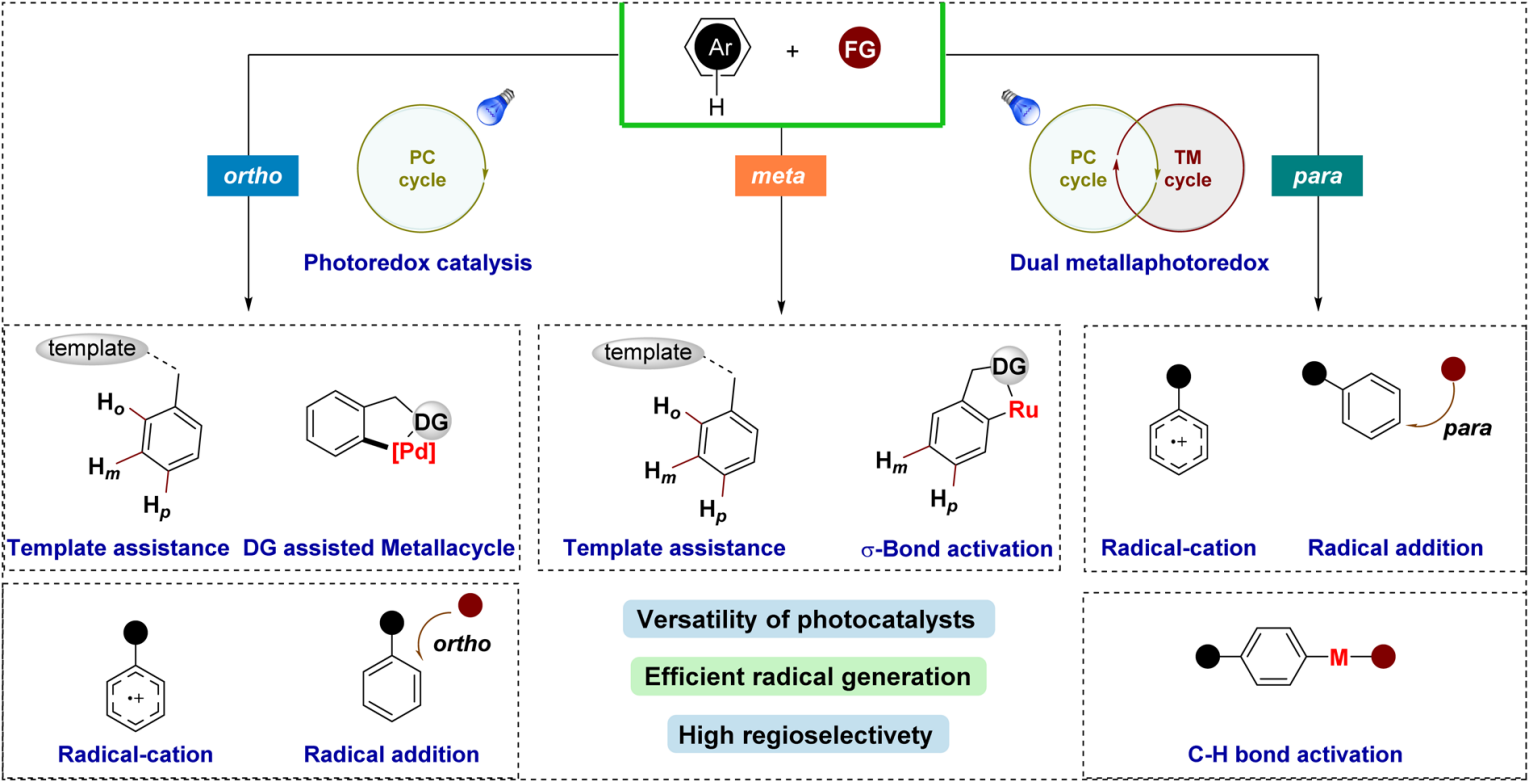
Summary of this review.

Several strategies have been developed to achieve regioselective functionalization of arenes *via* photocatalytic pathways (Fig. 2). One prominent approach is σ-bond activation-assisted C–H functionalization, where initial *ortho*-cycloruthenation plays a pivotal role in guiding selective radical coupling at the *meta*- or *para*-position. Another method employs templates or directing groups, which position the reactive metal center near the targeted C–H bond using a molecular bridge to control the geometry between the substrate and the catalyst. An alternative strategy involves the oxidation of aromatic compounds, followed by nucleophilic attack by the reagent to functionalize the phenyl ring. Moreover, regioselective functionalization of arenes can also be achieved by first generating a radical *via* SET, which then inserts into the phenyl ring in a regioselective manner. However, these latter methods often face challenges in achieving high regioselectivity, as the outcome is highly substrate-dependent.

Recent literature has extensively reviewed photocatalyzed aromatic C–H bond functionalization^11,31–33^ and regioselective C–H bond functionalization of arenes.^1,3,15,16^ However, comprehensive articles specifically addressing photocatalytic regioselective C–H bond functionalization are relatively scarce. Existing reviews predominantly focus on specific topics such as visible light-induced C–H functionalization^34^ with transition metal complexes or photoredox-catalyzed remote C–H bond functionalization.^35^ Despite significant advancements and detailed insights in these areas, there remains a need for a comprehensive review that systematically explores regioselectivity in photocatalytic phenyl C–H bond functionalization. This review aims to bridge this gap by offering an in-depth analysis of recent developments in the field, focusing on the regioselective photocatalytic C–H bond functionalization of benzene derivatives. It will assess the advantages, limitations, scopes, and reaction mechanisms of these transformations, providing critical insights to guide future research. The content is organized according to the site of functionalization on the benzene ring: *ortho-*, *meta-*, or *para*-positions, thus delivering a structured and detailed overview of regioselectivity in these processes.

## 2. *Ortho*-selective C–H functionalization

*Ortho*-C–H functionalization represents a pivotal transformation in organic synthesis, offering a direct approach to modify aromatic compounds without the need for pre-functionalization steps. This approach significantly streamlines synthetic pathways and enhances overall efficiency. Traditionally, these transformations required high temperatures and specific reagents, but recent advancements, including palladium-catalyzed methodologies and photoredox catalysis, have introduced milder conditions and expanded substrate compatibility.^10,34,35^ These innovations not only increase the versatility of C–H functionalization but also facilitate the incorporation of a wide range of functional groups into complex molecular architectures, thereby proving indispensable in drug discovery and materials science. Such *ortho*-regioselectivity can be achieved by using arenes containing heteroatom-based directing groups to facilitate *ortho*-selective reagent attack by coordinating with the catalyst or reagent. Alternatively, substrate control relies on carefully modulating electronic and steric factors inherent to the molecule, enabling site-selective functionalization predominantly at the *ortho*-position.

### 2.1. Directing group-mediated *ortho*-C–H functionalization

#### 2.1.1. *Ortho*-C–H arylation

Among the various C–H functionalization reactions, C–H arylation has gained substantial importance in materials science, crop protection, and pharmaceutical research.^36–38^ Notably, ruthenium-catalyzed C–H arylations have been employed to synthesize biologically active compounds such as anacetrapib, valsartan, and candesartan, with significant contributions from research groups at Merck, Ackermann, and Seki.^39–41^ Additionally, these ruthenium-catalyzed strategies have demonstrated considerable utility in the late-stage functionalization of peptides and nucleosides, broadening their application in the synthesis of complex molecules.^42,43^

**Scheme 1:**
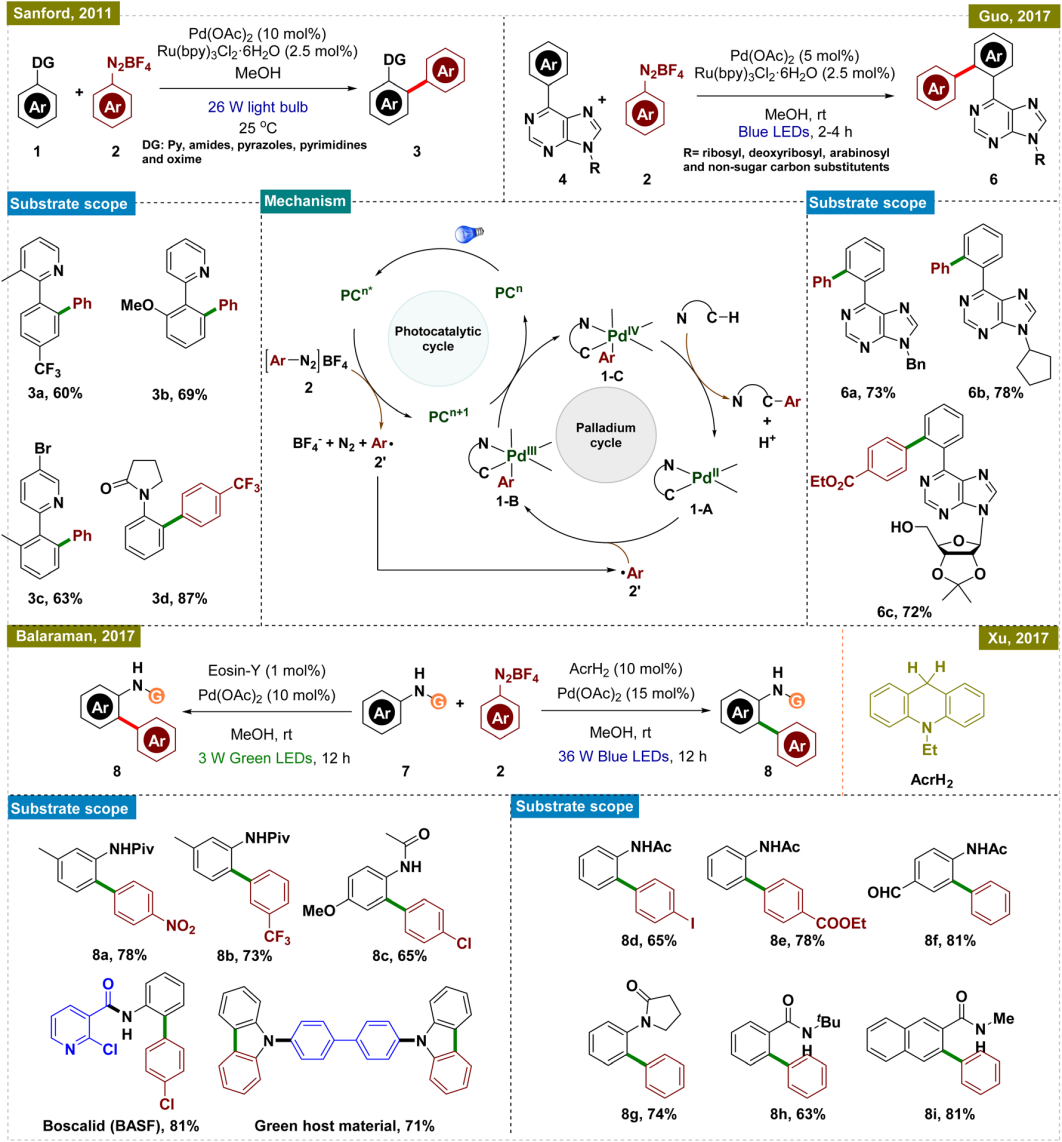
Photoinduced *ortho*-C–H arylation with aryldiazonium salts.

In this aspect, the group of Sanford reported a room-temperature ligand-directed C–H arylation that synergistically combined palladium-catalyzed C–H functionalization with visible-light photoredox catalysis (Scheme 1, top left).^44^ This protocol employed aryldiazonium salts as aryl radical precursors under ambient temperature, facilitating the efficient arylation of a wide range of substrates, whereas the previously reported conditions required a harsh reaction temperature of higher than 100 °C.^45–47^ These included pyridine derivatives with varied electronic properties as well as other directing groups such as amides, pyrazoles, pyrimidines, oxime ethers, and even free oximes. Although this report presented a significant advancement in the field of C–H arylation, the use of expensive Ru-based photocatalysts somewhat limited its application.

To address this drawback, research groups of Xu and Balaraman independently reported similar protocols for C(sp^2^)–H functionalization replacing Ru-photocatalysts with organo-photocatalysts, AcrH_2_ (9,10-dihydroacridine) and Eosin-Y, respectively (Scheme 1, bottom right and left).^48,49^ While their approach paralleled Sanford's work, they primarily employed amides as the directing group. Both protocols demonstrated a broad substrate scope, accommodating functional groups such as halides, esters, amides, and ketones, thereby extending their applicability to complex synthetic contexts. Notably, Balaraman's protocol was successfully utilized in the synthesis of boscalid, a potent fungicide and a green host material for phosphorescent OLEDs, highlighting its practical significance in both pharmaceuticals and advanced materials.

Guo and his group also utilized the same concept of dual catalysis for carrying out the *ortho*-C–H arylation of 6-arylpurine nucleosides, which are important motifs in medicinal chemistry due to their biological activities (Scheme 1, top right).^50^ By employing this dual catalytic protocol, they successfully arylated a variety of nucleosides by using aryl diazonium salts as arylating agents. Furthermore, the protocol was also applied to gram-scale synthesis, demonstrating its robustness and applicability for large-scale production.

To elucidate the reaction mechanism, a series of experimental studies were conducted, including radical trapping experiments and isotope effect analyses, independently in these reports. Based on these insights, a similar mechanism, as illustrated in Scheme 1, center, was proposed. The dual catalytic process was initiated by the photoexcitation of the photocatalyst (PC), which generated an aryl radical (2′) from the aryldiazonium salt (2) while concurrently getting oxidized to generate an oxidized PC (PC^*n*+1^). This aryl radical was then added to the palladacycle intermediate (1-A), which was formed *via* C–H activation of the substrate, leading to the formation of a Pd(iii) species (1-B). The Pd(iii) intermediate was further oxidized by the oxidized state of the photocatalyst (PC^*n*+1^), thereby regenerating the photocatalyst and producing a Pd(iv) intermediate (1-C). The catalytic cycle was concluded with a C–C bond-forming reductive elimination from the Pd(iv) intermediate, releasing the arylated product and regenerating the Pd(ii) catalyst, thus ensuring the continuity of the catalytic cycle. While these protocols offered significant advancements, they all necessitated the use of a photocatalyst in conjunction with a Pd catalyst for C–H activation, increasing the overall cost and limiting their practical applicability.

**Scheme 2:**
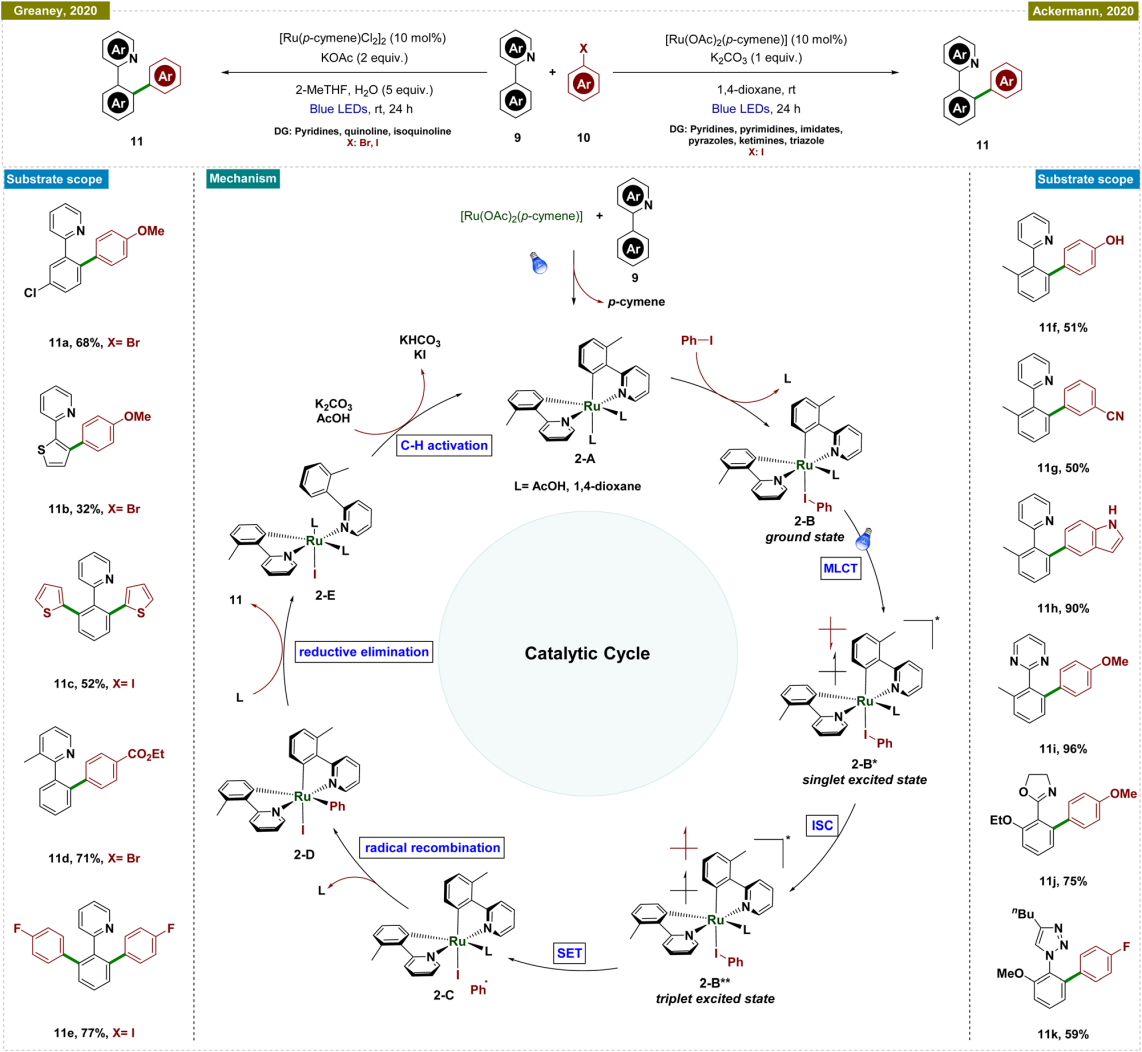
Photoinduced *ortho*-C–H arylation with aryl halides.

In contrast, Ackermann and Greaney groups reported an exogenous photocatalyst-free Ru-catalyzed C–H arylation by using aryl halides as effective aryl radical sources, which reduced the traditionally high reaction temperatures required for such processes (Scheme 2).^51,52^ Both of the research groups utilized a Ru-cymene-based catalyst; however, Greaney's team employed [Ru(*p*-cymene)Cl_2_]_2_, while Ackermann's team used [Ru(OAc)_2_(*p*-cymene)] as the catalyst. In both cases, a stoichiometric amount of base was necessary, and the reactions were conducted under the irradiation of blue LEDs. Greaney's protocol utilized various aryl bromides and iodides to arylate arenes with directing groups like pyridines, quinolines, and isoquinolines. Conversely, Ackermann's method was selective for aryl iodides, leaving bromides and pseudohalides such as -OTf intact, and was applicable to a broader range of directing groups, including pyridines, pyrimidines, imidates, pyrazoles, ketimines, and triazoles. Moreover, Ackermann's group extended their protocol to the late-stage diversification of biorelevant molecules such as purines, nucleosides, and nucleotides. Both groups conducted mechanistic studies, ruling out radical formation *via* quenching experiments and highlighting the critical role of the carboxylate ligand. Additionally, *p*-cymene was found to dissociate during the reaction, initiating the catalytic cycle. Detailed DFT studies by Ackermann's group further elucidated the mechanism, as depicted in Scheme 2.

The proposed catalytic cycle began with carboxylate-assisted C–H activation of 9 and *p*-cymene dissociation, forming the biscyclometalated complex 2-A (Scheme 2). Coordination of the haloarene to complex 2-A generated ruthenacycle 2-A, which, upon blue-light excitation, formed the singlet excited species 2-B*. After intersystem crossing (ISC) to a long-lived triplet state (2-B**), an inner-sphere electron transfer (ISET) occurred, producing a phenyl radical and a ruthenium(iii) intermediate (2-C). These recombined to yield a stable ruthenium(iv) species (2-D). Reductive elimination followed by ligand exchange released the arylated product and regenerated the active ruthenium(ii) complex (2-E), completing the cycle.

**Scheme 3:**
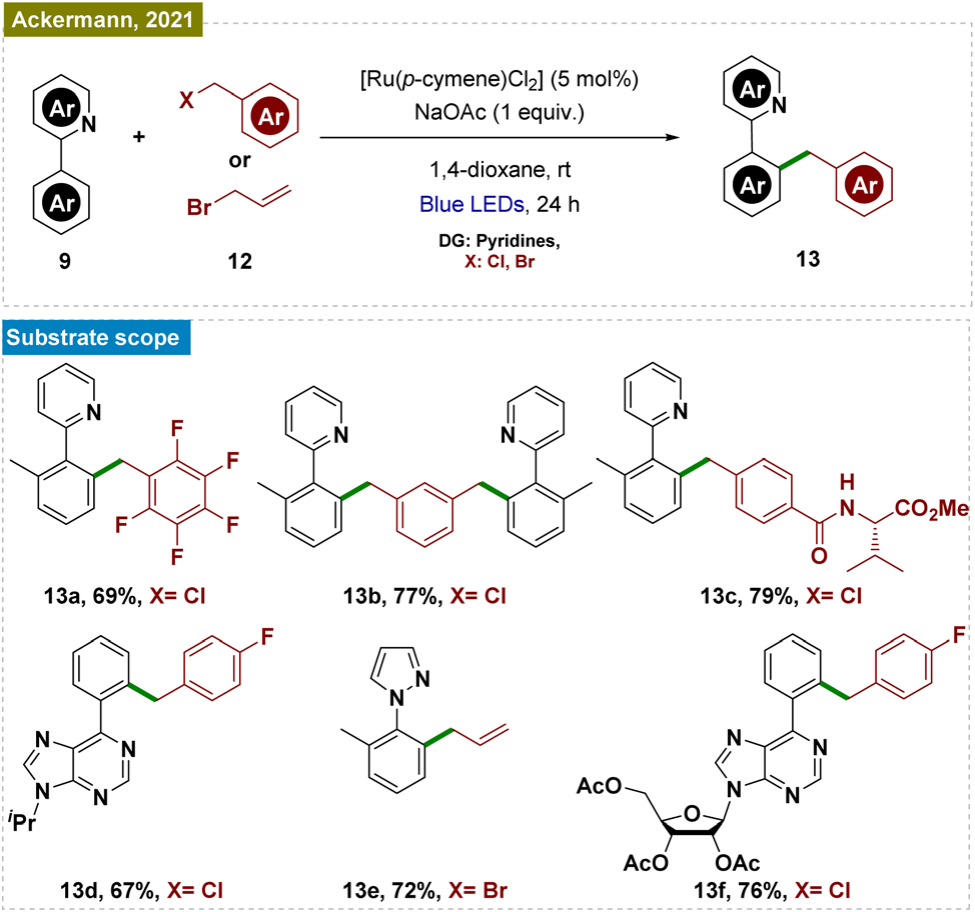
Photoinduced *ortho*-C–H benzylation and allylation.

Recently, Ackermann and his team also reported a Ru-catalyzed C–H benzylation and allylation reaction, which followed a similar mechanistic pathway (Scheme 3).^53^ This exogenous photocatalyst-free ruthenium catalysis was performed *via* a Ru(ii/iii) cycle, effectively functionalizing a range of arenes, including those with pyridines, pyrazoles, oxazolines, and biorelevant purines as directing groups, using various substituted benzyl chlorides and allyl bromides. The protocol also extended to electrophiles bearing biorelevant groups and even utilized water as a non-toxic solvent. Mechanistic studies and control experiments were conducted to substantiate the proposed mechanism, similar to that illustrated in Scheme 2.

#### 2.1.2. *Ortho*-C–H olefination

**Scheme 4:**
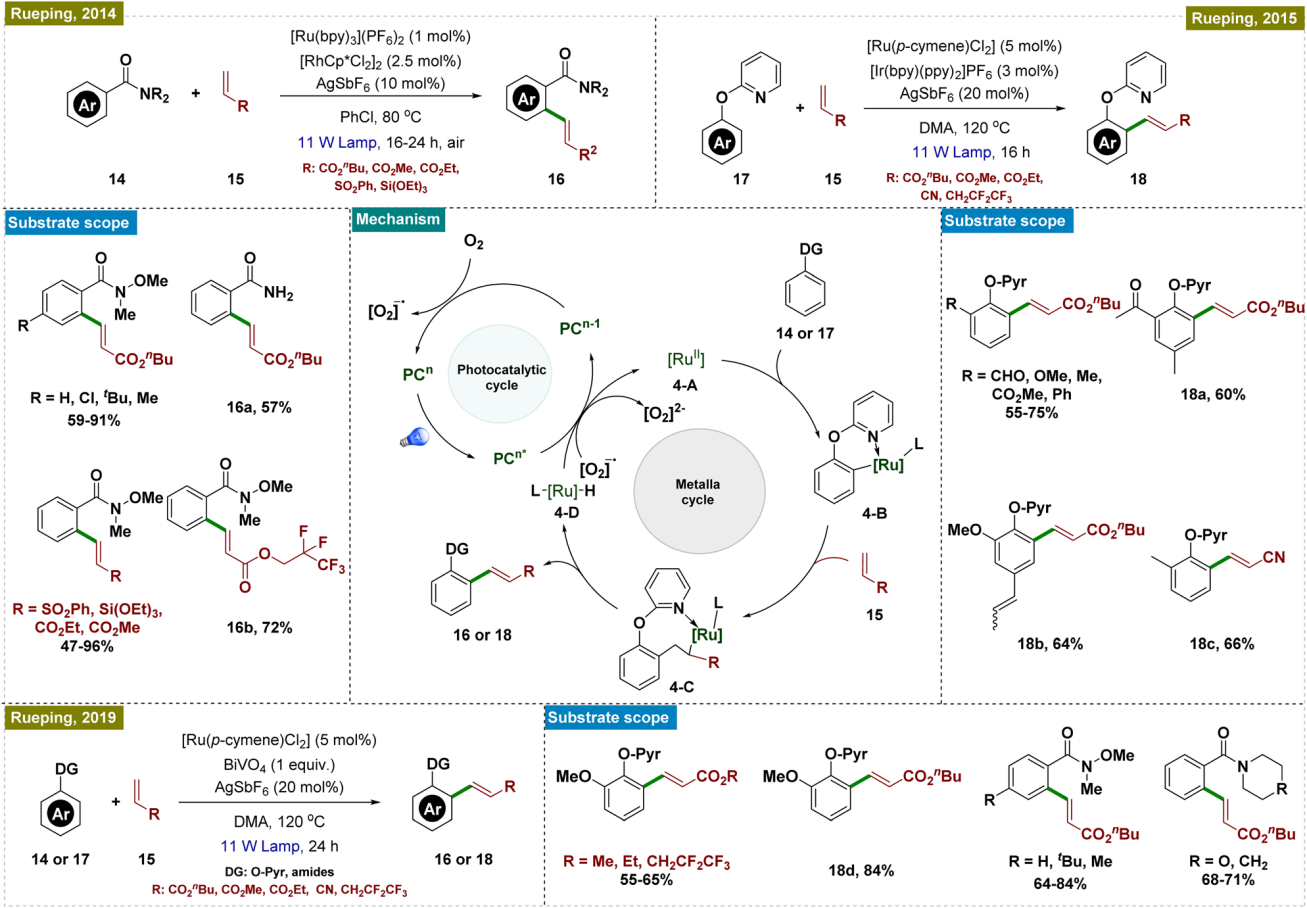
Photoinduced directed *ortho*-C–H olefination.

A rhodium-catalyzed *ortho*-C–H functionalization of aryl amides, leading to the formation of versatile building blocks,^54^*ortho*-olefinated Weinreb amides, was disclosed by the group of Rueping in 2014 (Scheme 4, top left).^55^ They successfully overcame the limitation of the requirement of external oxidants and harsh reaction conditions required for such reactions and performed the reaction at a much lower temperature.^56,57^ By leveraging a dual catalytic system combining photoredox and rhodium catalysis under visible light, the team achieved the direct oxidative C–H olefination of aryl amides. The methodology could be used to functionalize a range of aryl amides containing both electron-withdrawing as well as electron-donating substituents with various activated alkenes, including acrylates, vinyl sulfones, and silanes. Subsequently, the same research group further advanced their work by developing a photoredox-catalyzed C–H olefination of phenolic ethers, building upon Ackermann's previous findings.^58^ They employed an Ir-based photocatalyst in combination with a Ru-catalyst to achieve *ortho*-olefination of various phenol derivatives, specifically *ortho*-(2-pyridyl)phenols (pyr, see Scheme 4, top right).^59^ The regeneration of the ruthenium catalyst was facilitated through a photoredox-catalyzed oxidative process, enhancing the overall efficiency of the reaction.

Recently, they also employed semiconductors as heterogeneous photocatalysts in dual metal-catalyzed C–H functionalization reactions, including the *ortho*-olefination of phenol derivatives, the olefination of Weinreb amides, and indole synthesis (Scheme 4, bottom).^60^ In these processes, various metal catalysts were combined with semiconductor photocatalysts [(BiVO_4_ (band gap: 2.4 eV), WO_3_ (band gap: 2.6–3.0 eV), and TiO_2_)] to achieve the desired transformations.

A general mechanism for the directing group-assisted photocatalytic *ortho*-C–H olefination is depicted in Scheme 4. Upon the formation of the active Ru(ii) catalyst 4-A, which occurred by following the precipitation of chloride ligands from the Ru dimer, *ortho*-C–H activation of the substrate took place, leading to the formation of intermediate 4-B, facilitated by the coordinating directing group (shown with pyridine in Scheme 4). Subsequent olefin insertion produced intermediate 4-C, followed by reductive elimination to yield the desired product and generate the ruthenium complex 4-D. The photoredox catalyst, PC^*n*^, upon excitation by visible light, oxidized complex 4-D*via* electron transfer, thereby regenerating the catalytically active Ru species 4-A. The resulting PC^*n*−1^ was reoxidized to PC^*n*^ by molecular oxygen, generating a superoxide anion. This superoxide anion further oxidized Ru complex 4-D, accepting an electron and thus maintaining the catalytic cycle.

#### 2.1.3. *Ortho*-C–H acylation

**Scheme 5:**
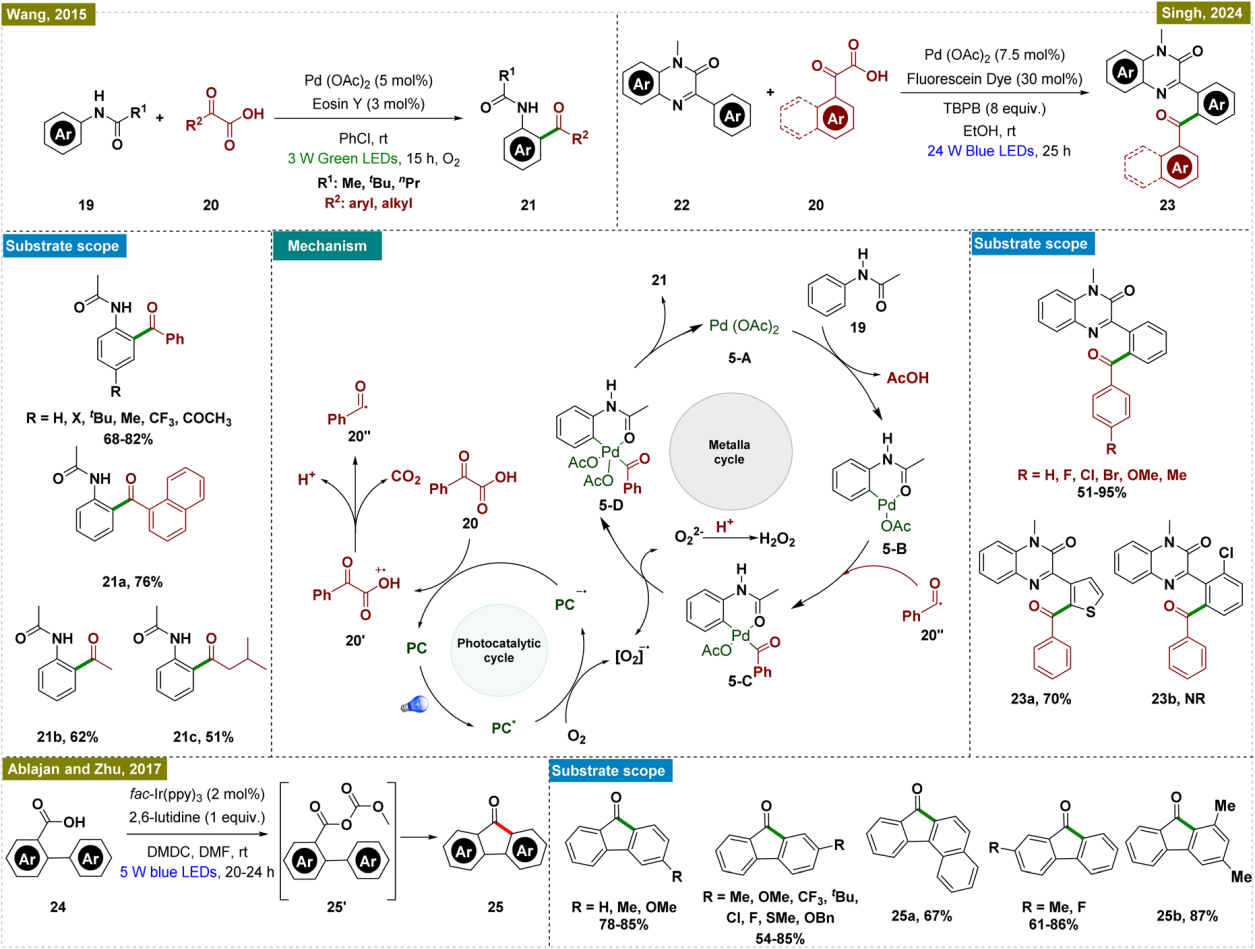
Photoinduced *ortho*-C–H acylation.

In 2015, Wang and co-workers presented a novel approach for the decarboxylative *ortho*-acylation of acetanilides by using α-oxocarboxylic acids, leveraging a dual catalytic system with Eosin Y as a photoredox catalyst and palladium acetate (Scheme 5, top left).^61^ Notably, this organic dye proved to be more cost-effective and practical compared to other transition metal-based photoredox catalysts. This method demonstrated significant advancements in synthetic chemistry, particularly through its mild reaction conditions, broad substrate scope, and high functional group tolerance, making it valuable for complex molecule synthesis in medicinal chemistry. Various mechanistic experiments, like radical trapping by TEMPO and ESR, to confirm the formation of a superoxide radical anion (O_2_˙^−^) were performed to elucidate the mechanism.

Based on the mechanistic and control experiments, a probable mechanism was proposed (Scheme 5, center). Initially, the photocatalyst (Eosin Y, PC) was excited to its excited state (PC*) under the irradiation of visible light. This excited state of the PC underwent an oxidation process with phenylglyoxylic acid (20), producing a benzoyl radical (20′′). Concurrently, the reduction of PC* generated the radical anion PC˙^−^. Electron transfer from PC˙^−^ to molecular oxygen regenerated the photocatalyst for the next catalytic cycle and formed a superoxide radical anion (O_2_˙^−^). Concurrently, the palladium catalytic cycle was initiated through C–H activation of acetanilide by Pd(OAc)_2_, forming a palladacycle intermediate (5-B). It is important to note that, in our view, the addition of substrate 19 to Pd(OAc)_2_ to form intermediate 5-B may have occurred through a photoinduced LMCT mechanism.^62^ This intermediate reacted with the *in situ*-generated benzoyl radical (20′′) to yield a Pd(iii) species (5-C), which was further oxidized by the superoxide radical anion to a Pd(iv) intermediate (5-D), with the concomitant formation of O_2_^2−^ and H_2_O_2_. The final step involved reductive elimination from the Pd(iv) intermediate, producing the desired *ortho*-acylated product and regenerating the Pd(ii) catalyst (5-A), thereby completing the catalytic cycle.

Building on a similar catalytic framework, Singh and colleagues recently developed a novel method for the regioselective decarboxylative acylation of *N*-methyl-3-phenylquinoxalin-2(1*H*)-ones (22) using dual palladium-photoredox catalysis under visible light (Scheme 5, top right).^63^ The method employed fluorescein dye as the photocatalyst and *tert*-butyl peroxybenzoate as the oxidant, though an oxidant was required in excess. While the reaction conditions tolerated a wide range of substituents, *ortho*-substituents on the aryl ring failed to yield the desired product (23b). To elucidate the mechanism, various control experiments, including radical trapping experiments, were conducted, indicating a mechanistic pathway similar to that proposed by Wang and co-workers.^61^

Continuing along similar lines, Ablajan, Zhu, and colleagues developed a novel method for synthesizing fluorenone derivatives (25) *via* dual photoredox-catalyzed deoxygenative intramolecular acylation reactions at room temperature (Scheme 5, bottom).^64^ Their protocol enabled the synthesis of a diverse array of fluorenones, featuring both electron-donating and electron-withdrawing substituents. This method represented a significant advancement over traditional fluorenone synthesis techniques, such as Friedel–Crafts acylations,^65,66^ fluorenes oxidations,^67,68^ and Diels–Alder reactions,^69^ which generated considerable waste. Through a series of mechanistic experiments and control studies, the researchers proposed a reaction mechanism aligned with previous reports, highlighting the crucial role of dimethyl dicarbonate (DMDC) and 2,6-lutidine in facilitating acyl radical formation. The study revealed that DMDC and 2,6-lutidine facilitate the formation of an anhydride intermediate (25′) from the biaryl carboxylic acid. This anhydride intermediate is essential for generating the acyl radical through a SET process. The resulting radical undergoes an intramolecular *ortho*-addition, ultimately yielding the desired product *via* a one-electron oxidation and deprotonation step.

#### 2.1.4. *Ortho*-C–H borylation

**Scheme 6:**
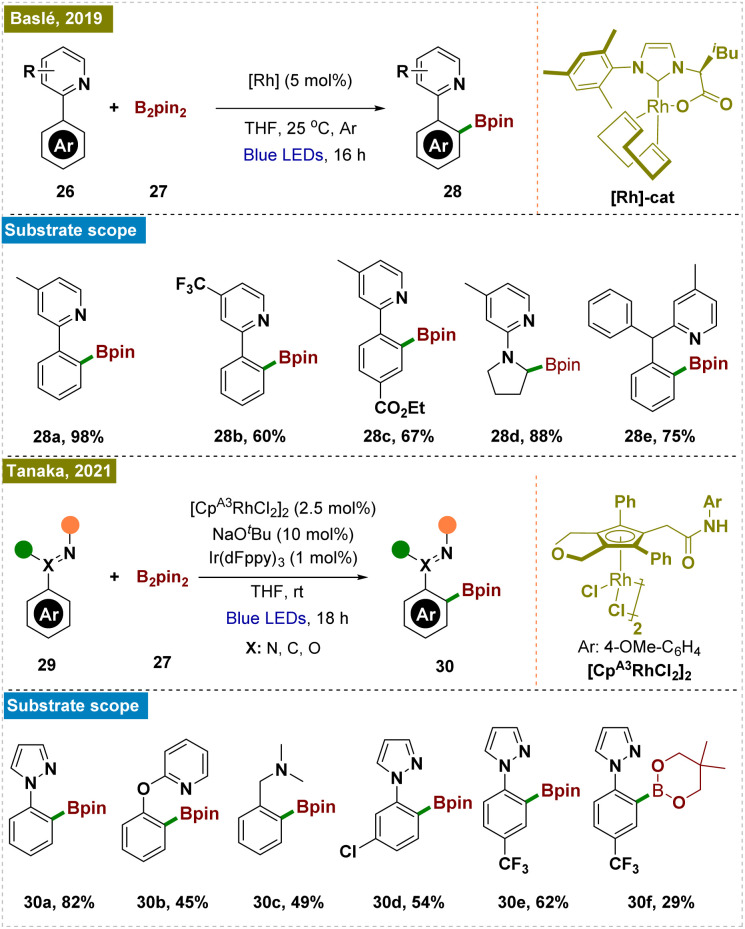
Photoinduced *ortho*-C–H borylation.

Organoboron compounds, which serve as highly versatile intermediates in medicinal chemistry and materials science, can be efficiently synthesized through the direct C–H borylation of arenes.^70–72^ In this context, Baslé and colleagues introduced a pioneering photocatalytic method that employed a rhodium(i) complex activated by visible light to achieve regioselective borylation of pyridine-substituted aromatic C–H bonds under mild, room-temperature conditions (Scheme 6, top).^73^ This approach leveraged a single N-heterocyclic carbene (NHC) rhodium(i) complex, which enhanced both the stability and reactivity of the catalyst. Consequently, this system enabled the efficient functionalization of a wide range of substrates, highlighting its broad applicability and potential in synthetic chemistry.

Later, Tanaka and colleagues reported a method for photo-induced *ortho*-C–H borylation of arenes through the *in situ* generation of rhodium(ii) ate complexes (Scheme 6, bottom).^74^ This study presented a novel approach to *ortho*-C–H borylation of arenes through the use of photoexcited rhodium complexes, specifically demonstrating the *in situ* generation of anionic rhodium(ii) ate complexes from rhodium(iii) precursors under blue LED irradiation. The catalyst optimization identified a CpRh(iii) complex with an electron-rich *N*-phenylcarbamoyl moiety as the most effective, achieving a 65% yield with NaO*t*Bu as the base. Mechanistic investigations suggested that the process involved the reduction of Rh(iii) to Rh(i), followed by oxidative addition to the *ortho*-C–H bond, leading to the formation of an aryl Rh(iii) hydride intermediate and ultimately yielding the borylated product. The method also expanded the scope of C–H functionalization by accommodating a broader range of directing groups. Computational studies using TDDFT calculations further corroborated the experimental observations, providing insight into the electronic transitions and photophysical properties of the rhodium complexes.

#### 2.1.5. *Ortho*-C–H alkynylation

**Scheme 7:**
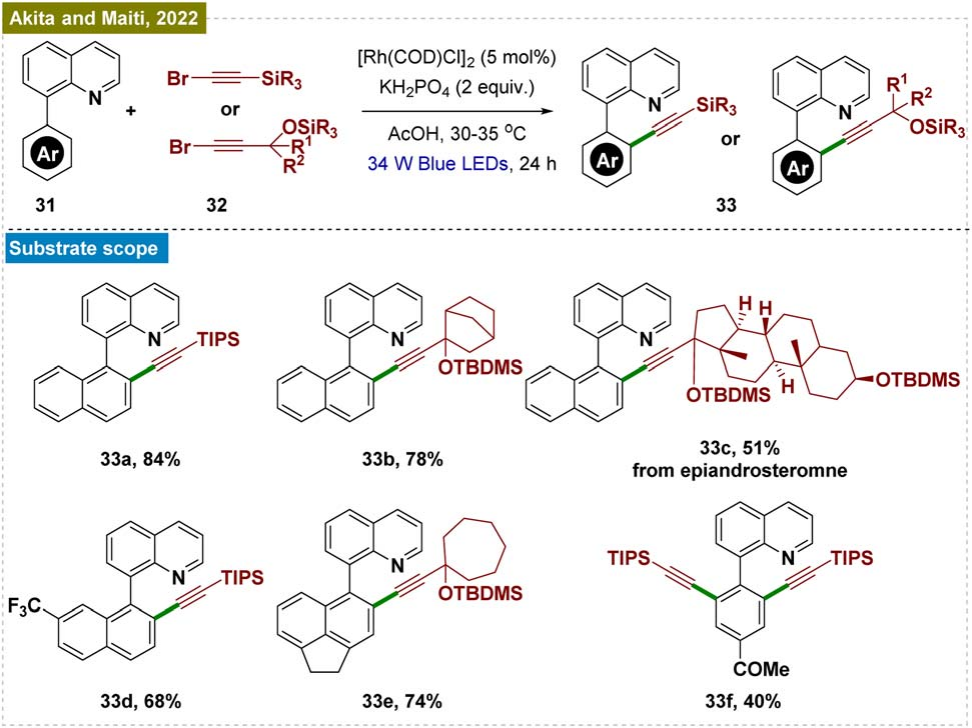
Photoinduced *ortho*-C–H alkynylation.

The introduction of alkynyl groups into arenes is of significant importance due to their potential for further functionalization and transformation.^75^ In light of this, Akita, Maiti, and colleagues have developed a Rh-catalyzed method for *ortho*-C–H alkynylation that was performed without the need for photocatalysts or silver,^76^ which are typically required for analogous thermally driven reactions (Scheme 7).^77–80^ This method was compatible with a variety of alkyne derivatives, enabling the incorporation of pharmacologically relevant molecules into arenes *via* an alkyne bridge. The process relied on the unique cooperative effect of a six-membered rhodacycle, identified as the photo-responsive species driving the transformation. Mechanistic and computational studies provided insights into the reaction pathway, suggesting that the photo-excited Rh species facilitated electron transfer to the *in situ* generated high-energy alkynyl radical through an outer-sphere mechanism, rather than *via* the more common oxidative addition or 1,2-migratory insertion routes. This work represented a significant advancement in the straightforward synthesis of alkynyl arenes, broadening the scope of C–H functionalization strategies.

### 2.2. Substrate-controlled *ortho*-C–H functionalization

#### 2.2.1. *Ortho*-C–H alkylation through the radical addition pathway

**Scheme 8:**
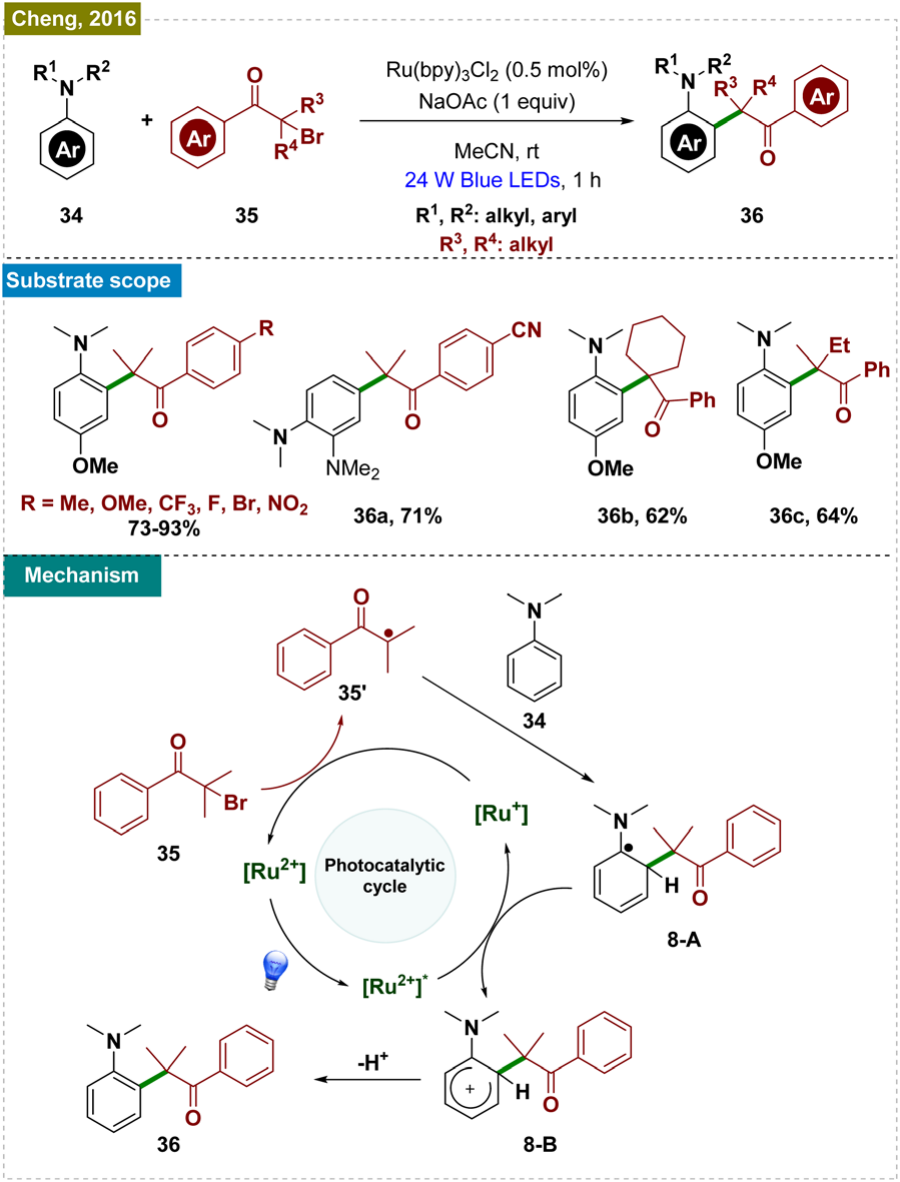
Photoinduced *ortho*-C–H alkylation from α-bromoketones.

Cheng and co-workers presented a visible-light-driven catalytic strategy for the intermolecular C–H quaternary alkylation of aniline derivatives by using α-bromo ketones (Scheme 8, top).^81^ This approach facilitated the efficient formation of quaternary carbon centers across a broad spectrum of functionalized substrates, including conjugated alkenes and other reactive groups. Remarkably, the reaction was also viable under natural sunlight and proved to be effective on a gram scale, underscoring its practical applicability. The study highlighted a regioselective preference for alkylation at the *ortho*-position relative to the amine group. This regioselectivity was rigorously examined by using DFT calculations of Fukui indices, spectroscopic analysis, and radical trapping experiments, providing deep insights into the mechanistic details of the radical addition process and emphasizing the enhanced reactivity of the *ortho*-position in relation to the amine group compared to the methyl or methoxy group. The scope of this approach is, however, notably constrained, as it necessitates the presence of a *para*-substituent to achieve effective *ortho*-regioselectivity. In its absence, as demonstrated with substrate 36a, the reaction predominantly yields the *para*-functionalized product. The reaction was initiated by the excitation of the ruthenium catalyst, which then entered the catalytic cycle. A SET event from Ru(i) to α-bromo isobutyrophenone (35) induced mesolytic cleavage, generating the isobutyrophenone radical 35′. The radical 35′ subsequently adds to aniline derivatives (34), forming the neutral radical species 8-A. This intermediate was then oxidized by the excited Ru(ii), leading to the formation of the cationic intermediate 8-B. The final deprotonation step yielded the desired product (Scheme 8, bottom). However, an alternative mechanism could involve the formation of radical cations from the aniline derivatives (*via* Ru^2+^ to Ru^1+^), followed by radical–radical coupling with the generated radical species 35′ (*via* Ru^1+^ to Ru^2+^), which may also produce the desired product.

#### 2.2.2. *Ortho*-C–H trifluoromethylation through the radical addition pathway

**Scheme 9:**
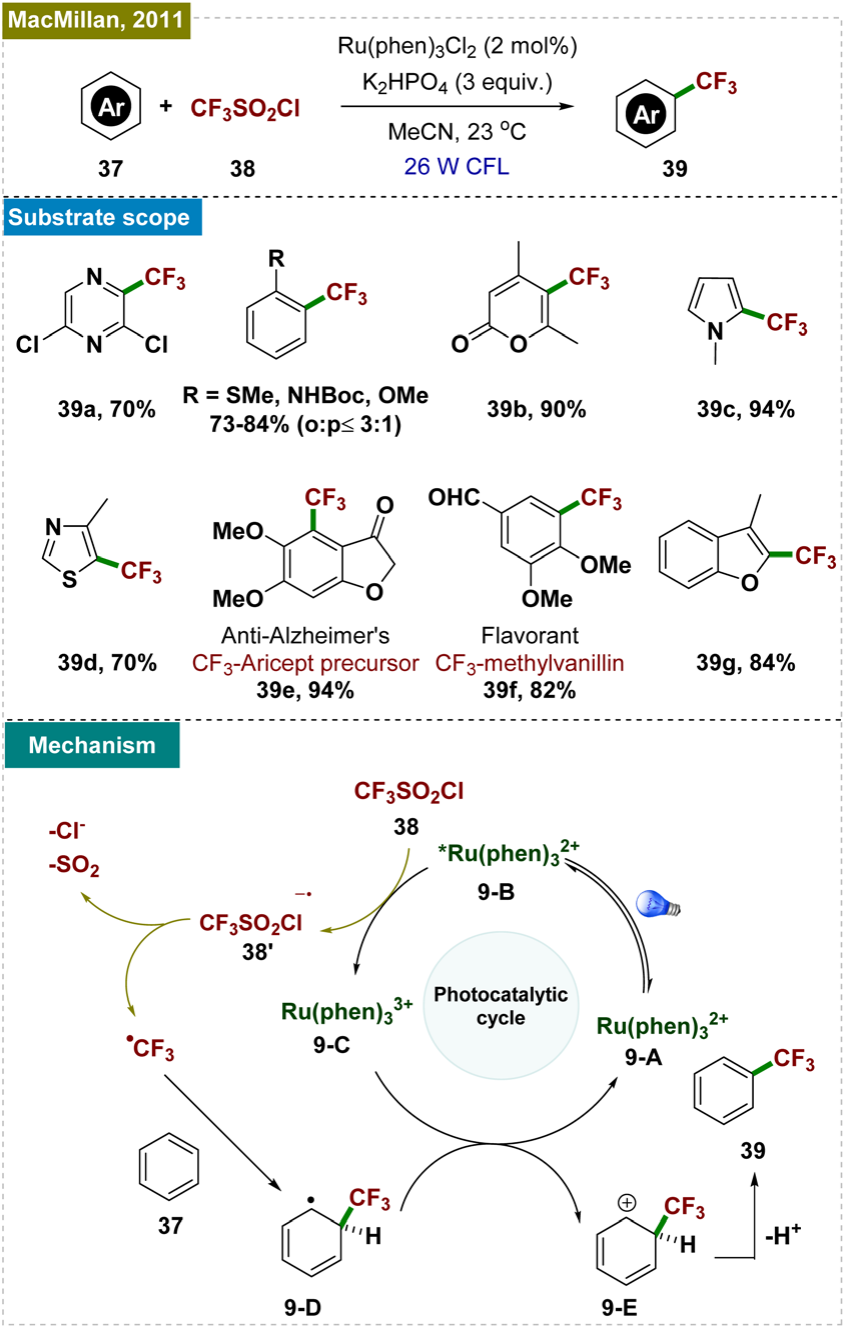
Photoinduced *ortho*-C–H trifluoromethylation.

Trifluoromethylation is a critical transformation in medicinal chemistry, as the incorporation of a –CF_3_ group into pharmaceutical agents significantly enhances their bioactivity.^82–84^ Addressing the necessity of more efficient methodologies, Nagib and MacMillan developed a radical-mediated trifluoromethylation of various arenes, employing a Ru-based photocatalyst (Scheme 9).^85^ Unlike previous methods that required harsh conditions, this approach utilized trifluoromethane sulfonyl chloride, a cost-effective and user-friendly reagent. Their protocol was applied for the trifluoromethylation of various (hetero)arenes, including some biologically active molecules. While the protocol demonstrated regioselectivity with a preference for functionalization at the *ortho*-position, the formation of the *para*-product was inevitable under these conditions. This outcome contrasts with the *para*-selective trifluoromethylation reported by Shi and Zhao,^86^ as detailed in Section 4.1, where using an organic photocatalyst preferentially facilitated *para*-selective functionalization.

Through quenching experiments and mechanistic studies, the authors proposed the mechanism, as depicted in Scheme 9. The reaction began with the photoexcitation of the photocatalyst (9-A), leading to its excited state (9-B). A single-electron transfer (SET) event from the excited state of the PC to triflyl chloride (38) generated the oxidized Ru^3+^ photocatalyst (9-C) and a –CF_3_ radical, which rapidly reacted with the arene (37) to form a cyclohexadienyl radical intermediate (9-D). This intermediate underwent a second SET with the oxidized Ru^3+^ photocatalyst, regenerating the active catalyst (9-A) and cyclohexadienyl cation (9-E). The final step involved the deprotonation of the cyclohexadienyl cation in the presence of a base, yielding the desired trifluoromethylated product. This method offered a milder, more accessible approach to trifluoromethylation, broadening its applicability in synthetic organic chemistry.

#### 2.2.3. *Ortho*-C–H phosphonylation through the radical cation pathway

**Scheme 10:**
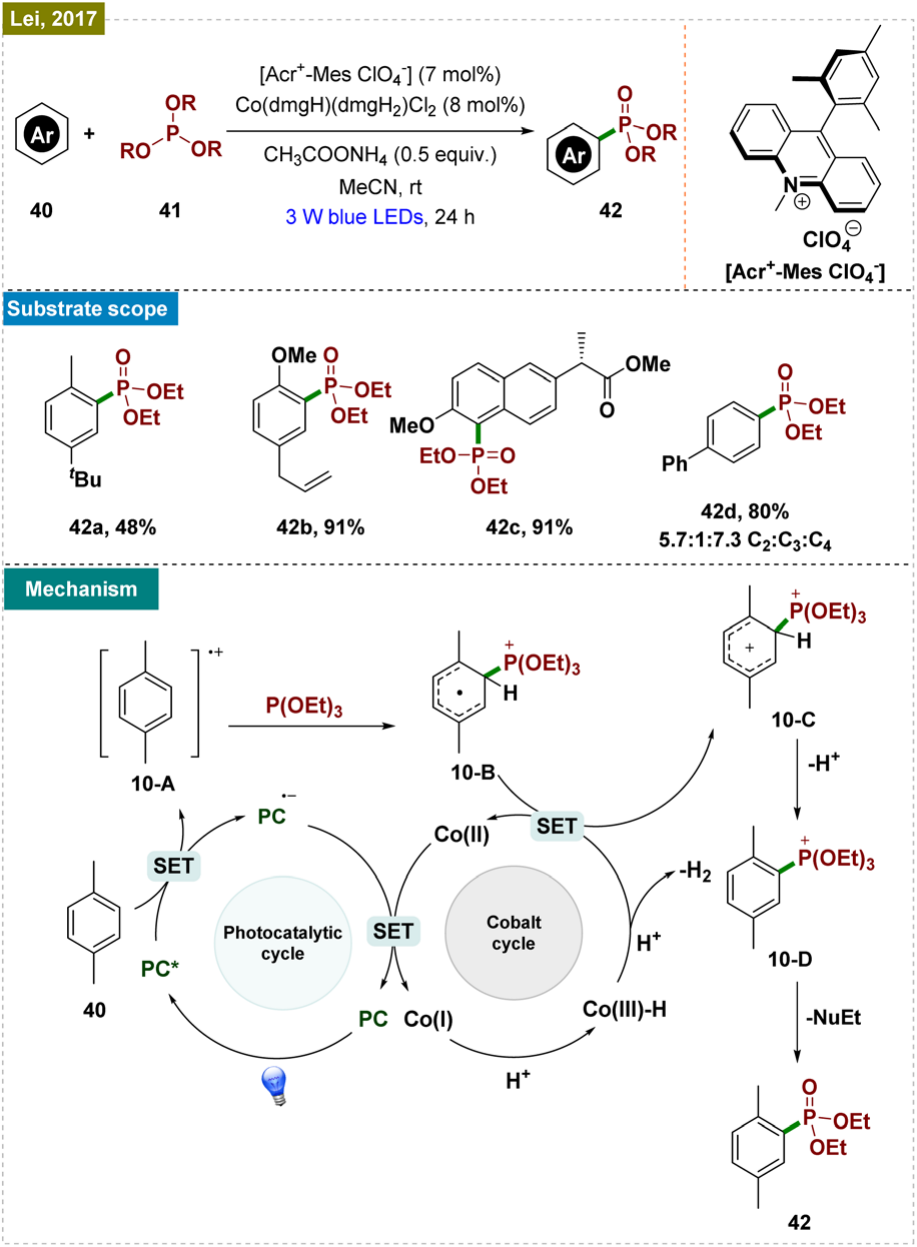
Photoinduced *ortho*-C–H phosphonylation.

Given the high synthetic value of phosphonates, the development of mild and sustainable catalytic phosphorylation strategies is of significant interest.^87–89^ Previous methods, though effective in achieving good regioselectivity, were often constrained by the necessity of directing groups and preactivation of substrates.^90^ Considering these limitations, Lei and colleagues introduced an innovative visible-light-induced, external oxidant-free oxidative phosphonylation of C(sp^2^)–H bonds, utilizing a dual catalytic approach that combined photocatalysis with proton-reduction catalysis.^91^ Their method employed Acr^+^-MesClO_4_^−^ as the photocatalyst, in synergy with the cocatalyst [Co(dmgH)(dmgH_2_)]Cl_2_ (Scheme 10). This strategy successfully enabled the synthesis of a diverse array of phosphonylated products and was further demonstrated through the late-stage functionalization of pharmaceutical compounds, highlighting its synthetic utility. However, the protocol exhibited substrate specificity, typically requiring the *para*-position to be blocked; otherwise, it consistently resulted in a mixture of regioisomers (42d). Extensive mechanistic studies were conducted to elucidate the reaction pathway, as outlined in Scheme 10. The reaction is initiated with the oxidation of the substrate (40) by the excited state of the photocatalyst (PC*), generating the arene radical cation 10-A. Triethyl phosphite (P(OEt)_3_) then acted as a nucleophile, capturing this radical cation and forming intermediate 10-B. Concurrently, the Co(iii) catalyst underwent reduction to Co(ii) *via* single-electron transfer (SET), leading to the formation of arene intermediate 10-C. This intermediate, upon deprotonation, yielded the phosphonylated intermediate 10-D. The additive CH_3_COONH_4_ facilitated the conversion of the arylphosphonium salt into the final phosphonylated product (42). The PC is regenerated through the oxidation of PC˙^−^ by Co(ii), which generates Co(i). The subsequent protonation of Co(i) generates Co(iii)–H, which undergoes further protonation to release H_2,_ thus closing the catalytic cycle.

#### 2.2.4. C–H nitration through the radical cation pathway

**Scheme 11:**
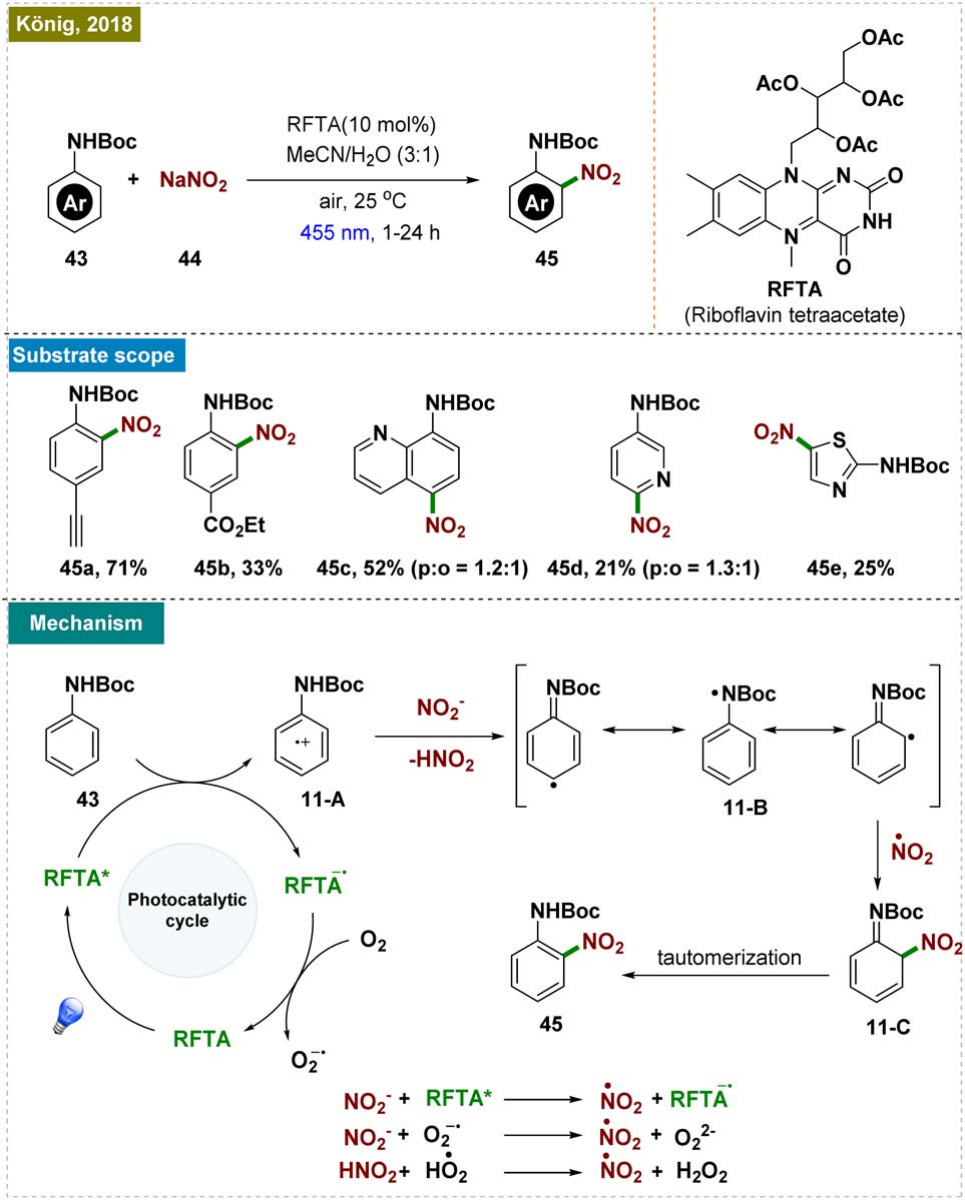
Photoinduced *ortho*-C–H nitration.

Nitroanilines represent another important class of compounds with significant applications in pharmaceuticals and dyes.^92^ Owing to this, various nitration protocols have been developed, although they required harsher conditions, such as strong acids and high temperatures. Although milder nitrating agents like *tert*-butyl nitrite (TBN) have been employed, these reactions still required elevated temperatures.^93–97^ In contrast to this, König and colleagues recently developed a method for synthesizing protected anilines using an organic photoredox catalyst and sodium nitrite as a cost-effective –NO_2_ source at room temperature (Scheme 11).^98^ Their protocol was applicable to a wide range of aniline derivatives, including substituents like alkynes and esters. However, a mixture of regioisomers, as seen in substrate 45d, was consistently observed. It is important to note that the regioselectivity is substrate-specific, governed by both the position and electronic properties of the substituent. In all cases, the *para*-functionalized product was the major regioisomer, although a mixture of regioisomers was obtained.

As illustrated in Scheme 11, the reaction mechanism began with the excitation of the photocatalyst, which oxidized the aniline derivative (43), generating the arene radical cation 11-A. Subsequent deprotonation led to the formation of intermediate 11-B, which, upon reacting with a nitrate radical, yielded the desired product *via* the intermediate 11-C.

#### 2.2.5. Heterocycles synthesized *via* intramolecular *ortho*-C–H functionalization

##### 2.2.5.1. Synthesis of benzothiazoles by *ortho*-C–H thiolation

**Scheme 12:**
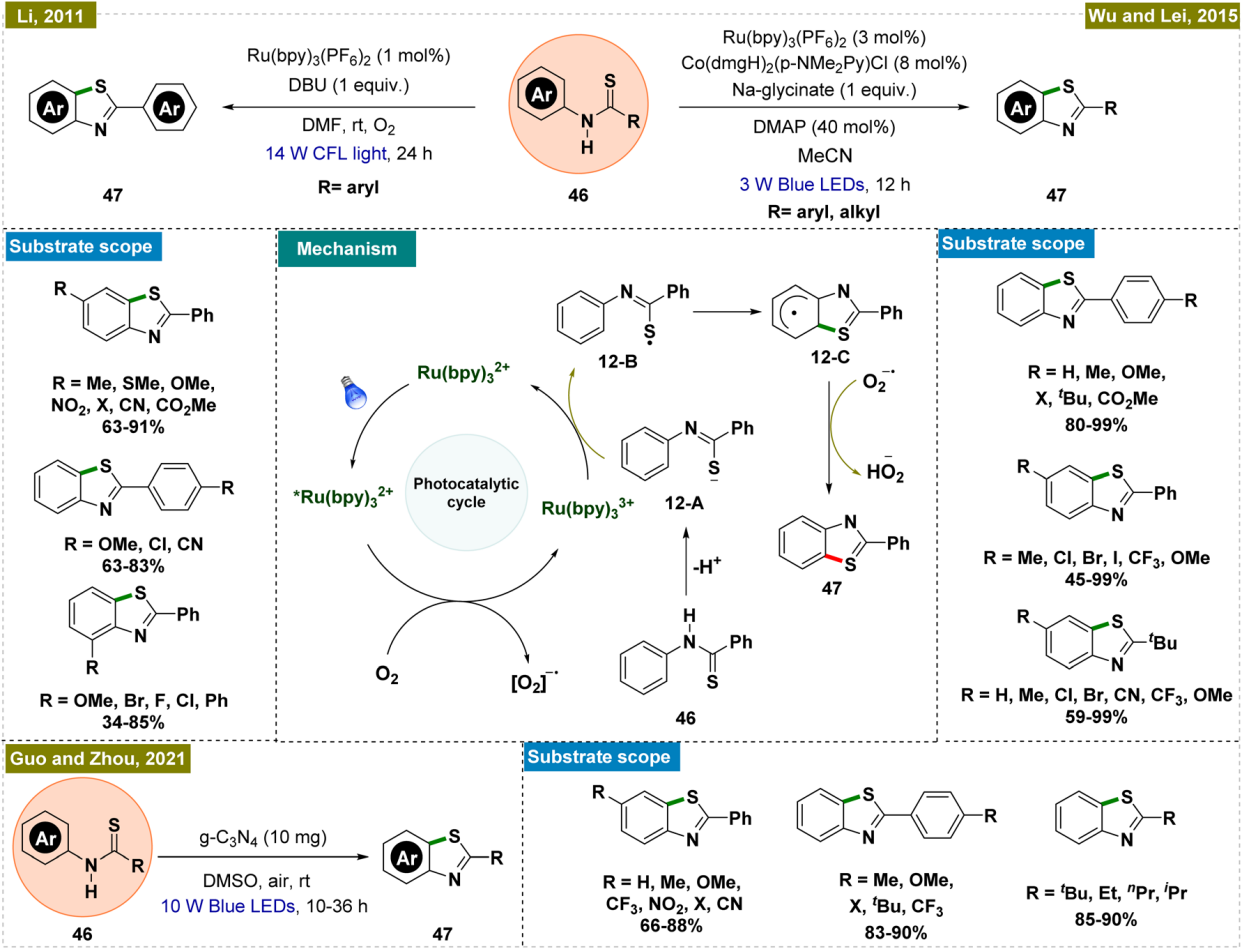
Synthesis of benzothiazoles by photoinduced *ortho*-C–H thiolation.

Benzothiazoles represent a crucial class of compounds with diverse biological and pharmaceutical applications.^99,100^ Considering this, in 2011, Li and colleagues introduced a mild and efficient methodology for synthesizing benzothiazoles through C–H functionalization, which was notable for its avoidance of direct metal catalysts and its use of visible light-mediated photoredox catalysis (Scheme 12, top left).^101^ This approach allowed for the effective synthesis of a variety of benzothiazole derivatives, with electron-donating groups showing enhanced reactivity. The reaction employed molecular oxygen as an oxidant and produced water as the sole byproduct, aligning with the principles of green chemistry. Mechanistic insights were gained through various control and kinetic experiments, leading to the proposed mechanism depicted in Scheme 12, center. Upon irradiation with light, the Ru(bpy)_3_^2+^ complex was excited to the *Ru(bpy)_3_^2+^ state. This excited photocatalyst was subsequently oxidized by molecular oxygen, producing the Ru(bpy)_3_^3+^ species and a superoxide radical anion (O_2_˙^−^) *via* a single electron transfer (SET) process. Concurrently, the thioanilide substrate (46) was deprotonated to form an anionic intermediate (12-A), which was then reduced to a sulfur-centered radical (12-B) through SET with the oxidized Ru(bpy)_3_^3+^, thereby regenerating the Ru(bpy)_3_^2+^ catalyst and completing the photoredox cycle. The sulfur-centered radical underwent cyclization *via* a nucleophilic attack on the benzene ring, forming a key intermediate (12-C). This intermediate subsequently transferred a hydrogen atom to the O_2_˙^−^ radical anion, facilitating rearomatization and resulting in the formation of the final benzothiazole product.

Wu and Lei subsequently further advanced the protocol by developing an external oxidant-free dual photoredox catalytic system for synthesizing benzothiazoles,^102^ marking a significant improvement over previous methods that often led to undesirable side products and necessitated the use of oxidants (Scheme 12, top right).^103,104^ Their approach was notably more efficient, producing only hydrogen as a byproduct and proceeding under oxidant-free conditions, although a base was required for the reaction. For the synthesis of 2-aryl-substituted benzothiazoles, a mild base such as sodium glycinate was sufficient, whereas the synthesis of 2-alkyl-substituted benzothiazoles required a stronger base like tetrabutylammonium hydroxide (TBAOH). Mechanistically, this method differed from Li's protocol,^101^ as it did not rely on molecular oxygen as the oxidant. Instead, the cobalt catalytic cycle played a crucial role in regenerating the photocatalyst and forming the cationic complex, facilitating the desired transformation.

Building on the principles established by Li's group,^101^ Guo and Zhou's team recently developed a novel heterogeneous catalytic system for intramolecular *ortho*-C–H thiolation to synthesize benzothiazoles (Scheme 12, bottom).^105^ This method employed a metal-free, heterogeneous photocatalyst based on graphitic carbon nitride (g-C_3_N_4_), enabling the efficient synthesis of a wide array of 2-substituted benzothiazoles, accommodating both aryl and alkyl substituents. The reaction proceeded under ambient air without the necessity of an additional base or oxidant. The simplicity of the system, coupled with the stability and reusability of the g-C_3_N_4_ catalyst—up to five cycles—made this approach highly practical and provided additional advantages over previous reports. Mechanistic and control studies suggested that substrate absorption onto g-C_3_N_4_ played a crucial role in facilitating the photocatalytic cycle and subsequent product formation, underscoring the catalyst's effectiveness in this transformation.

##### 2.2.5.2. Synthesis of indoles and coumarins by *ortho*-C–H cyclization

**Scheme 13:**
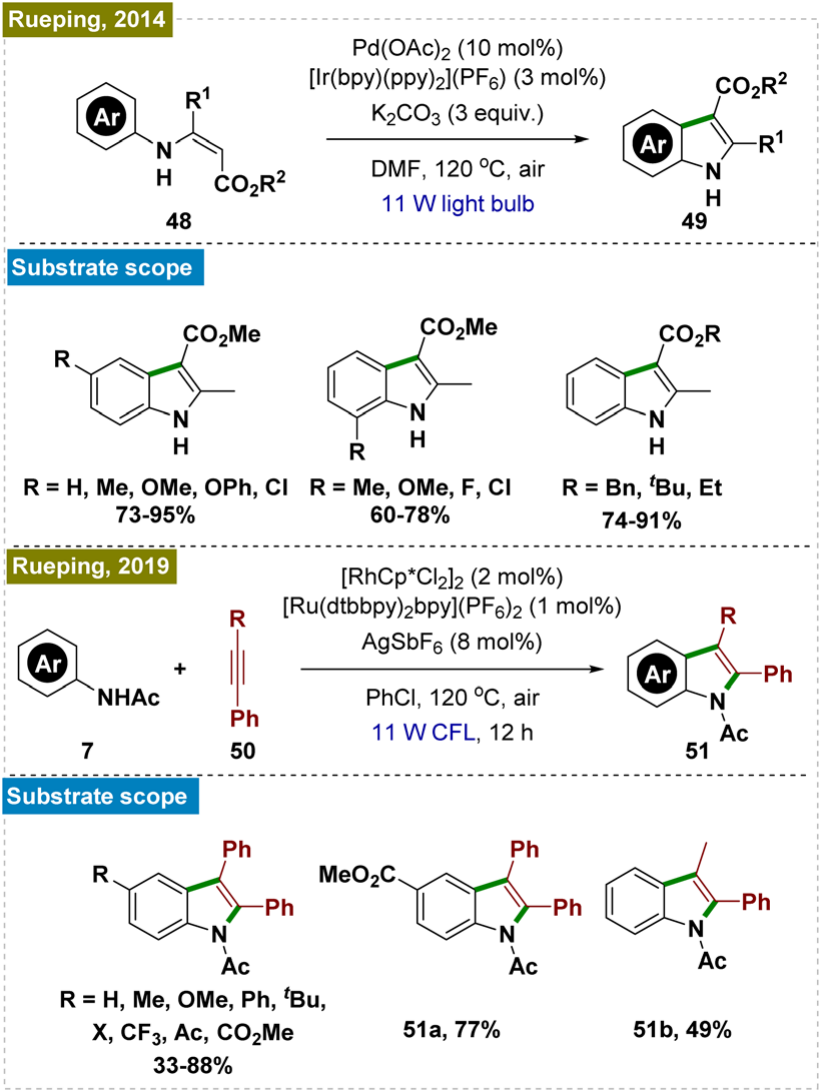
Synthesis of indoles by *ortho*-C–H cyclization.

Indoles and coumarins are pivotal compounds found in a variety of bioactive natural products, which has spurred significant research into the efficient construction of these important scaffolds.^106–109^ In this context, Rueping and his team developed a dual photocatalytic system for synthesizing a broad range of indoles through the intramolecular cyclization of aromatic enamines (Scheme 13, top).^110^ Although high temperature was still necessary, they successfully overcame the necessity of excess Cu(OAc)_2_—commonly used as an oxidant in earlier methods for regenerating active Pd catalysts—by employing a catalytic amount of an Ir-based photocatalyst.^111–113^

Subsequently, the same group introduced an alternative protocol for the synthesis of indoles *via* the cyclization of substituted anilides with alkynes (Scheme 13, bottom).^114^ To avoid the use of copper salts as oxidants, they used a Ru photocatalyst in conjunction with a Rh catalyst.^115,116^ Furthermore, they demonstrated that the reaction could be performed by using a heterogeneous inorganic photocatalyst (BiVO_4_) instead of the Ru photocatalyst. Mechanistic investigations indicated that the photoredox catalysis process proceeded independently of C–H activation, underscoring the crucial role of photoredox catalysts in generating superoxide radicals, which drove the reaction forward. This work highlighted the advances in indole synthesis and the broader potential of photoredox catalysis in organic synthesis.

**Scheme 14:**
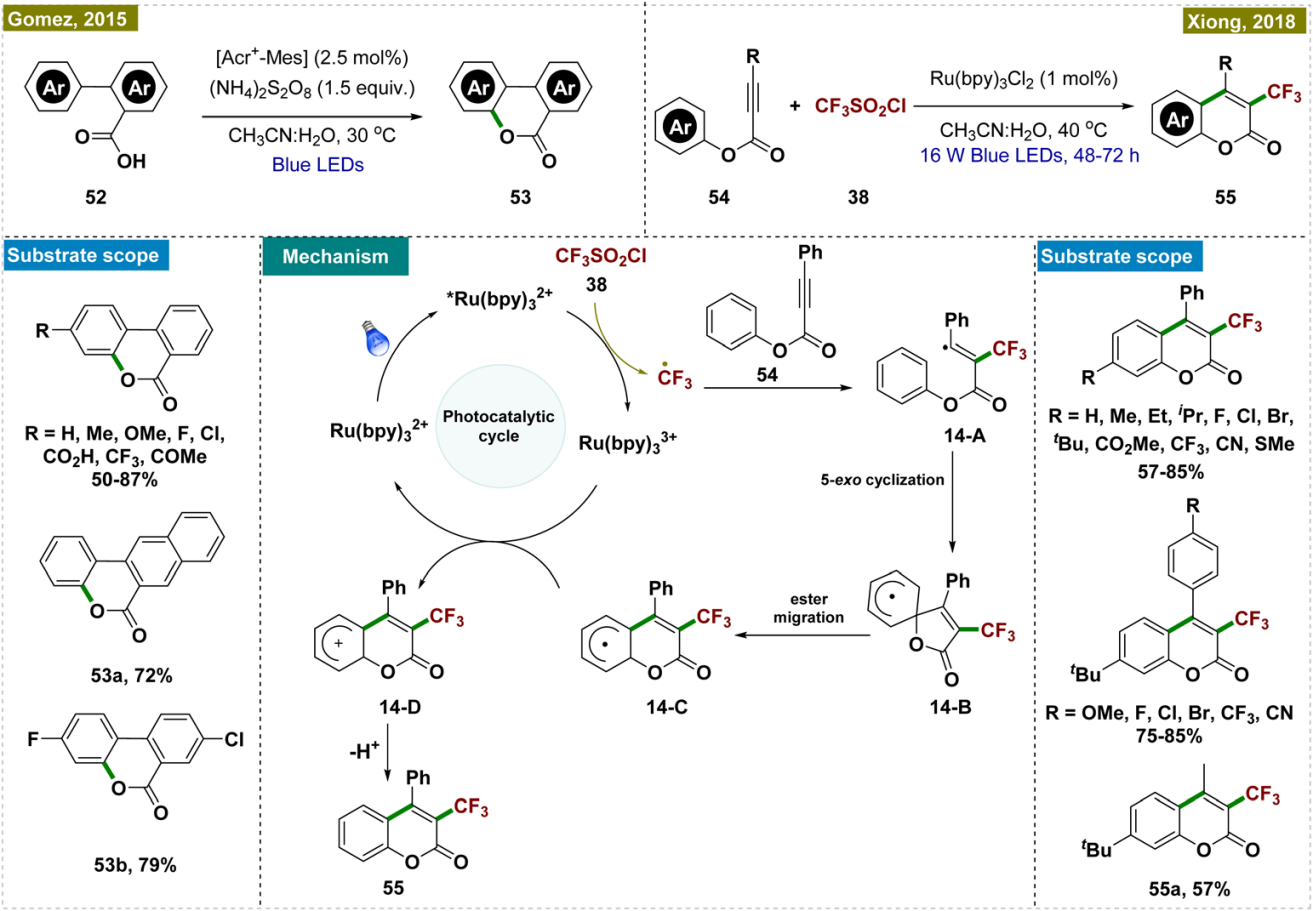
Synthesis of coumarins by *ortho*-C–H cyclization.

Gonzalez-Gomez and co-workers developed a novel protocol for synthesizing benzo-3,4-coumarins,^117^ a crucial intermediate in the synthesis of various natural products, which has been previously synthesized following harsh reaction conditions.^24,118,119^ Their approach utilized an organophotocatalyst, [Acr^+^-Mes], in combination with an excess of ammonium persulfate ((NH_4_)_2_S_2_O_8_) as the oxidant to facilitate the dehydrogenative lactonization of aryl benzoic acids (Scheme 14, top left). Crucially, no product formation was observed in the absence of either light or the photoredox catalyst, highlighting the indispensable role of photocatalysis in this transformation. Further mechanistic studies, including kinetic studies, were conducted to elucidate the mechanism.

Later, Xiong and co-workers developed an alternative protocol for the synthesis of 3-trifluoromethyl coumarins *via* a visible light-promoted cascade radical cyclization, employing CF_3_SO_2_Cl and Ru(bpy)_3_Cl_2_ as photocatalysts (Scheme 14, top right).^120^ This method offered milder reaction conditions compared to previous approaches that required more stringent conditions.^121^ Through various control experiments and mechanistic studies, the authors proposed a detailed reaction mechanism (depicted in Scheme 14, center). The process began with a single electron transfer (SET) reduction of triflyl chloride (38), coupled with the oxidation of *[Ru(bpy)_3_]^2+^ to [Ru(bpy)_3_]^3+^ under the irradiation of visible light, generating a trifluoromethyl radical. This radical then underwent electrophilic addition to *p*-methylphenyl 3-phenylpropiolate (54), forming intermediate 14-A, which subsequently transformed into radical 14-B through a 5-exocyclization. The next step involved the conversion of radical 14-B into radical 14-C*via* a 1,2-ester migration, a process previously observed in the literature.

While authors have documented the formation of 14-C through 5-*exo*-cyclization involving intermediate 14-B, an alternative phenomenon may occur, allowing the direct conversion of 14-A into 14-C*via* 6-*exo*-dig cyclization. Radical intermediate 14-C then underwent a second SET event with the strongly oxidizing Ru^3+^ photocatalyst, resulting in cationic intermediate 14-D. Finally, a proton was released and rearomatization yielded the desired product.

##### 2.2.5.3. Carbazole synthesis by intramolecular C–H amination

**Scheme 15:**
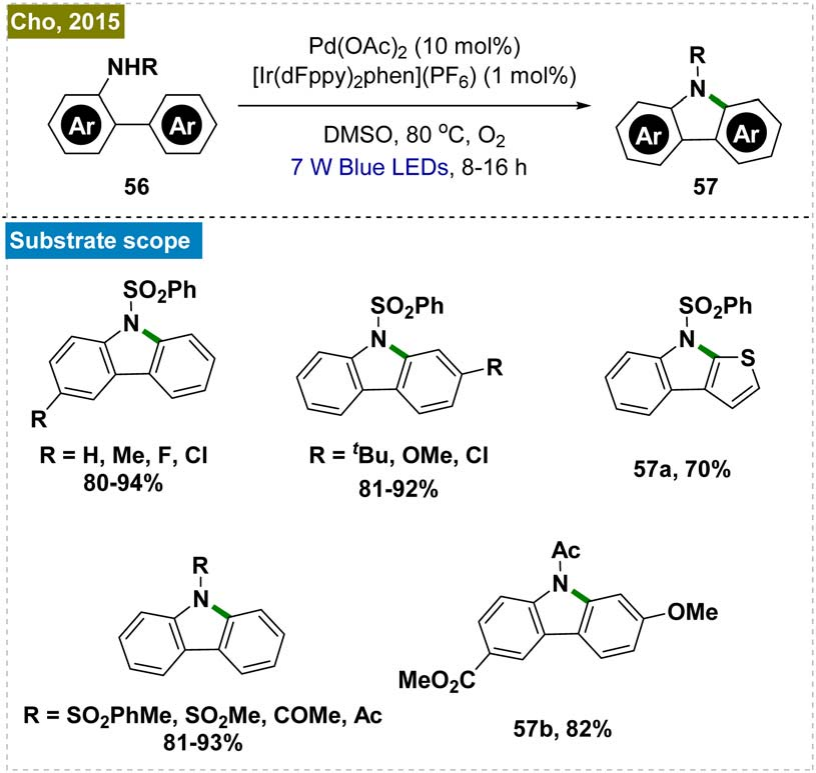
Synthesis of carbazoles by intramolecular *ortho*-C–H amination.

The synthesis of carbazoles, which are biologically significant molecules, traditionally necessitates high temperatures and the use of stoichiometric amounts of oxidants.^122–124^ To address these limitations, Cho and his team developed a dual photoredox-catalyzed intramolecular C–H amination of *N*-substituted 2-amidobiaryls, utilizing a combination of an Ir-based photocatalyst and Pd(OAc)_2_ under aerobic conditions (Scheme 15).^125^ This method was proved to be effective for synthesizing a diverse array of carbazole derivatives with various substituents. Notably, one of the carbazoles synthesized through this protocol was further transformed into the alkaloid clausine C in a single deprotection step.

## 3. *Meta*-selective C–H functionalization

*Meta*-selective C–H functionalization has been relatively underexplored compared to the well-established *ortho*- and *para*-functionalization strategies. This discrepancy arises from the intrinsic electronic properties of the benzene ring. In electron-deficient arenes, the *meta*-position becomes more reactive, but activating these electron-poor substrates remains challenging, particularly in the context of photocatalysis, where their lower reactivity presents a significant obstacle. Recent advances have sought to overcome these limitations through the development of novel methodologies for *meta*-selective C–H bond activation.

Approaches such as template-directed functionalization and σ-activation have shown promise in achieving *meta*-selectivity.^1^ These methods exploit weak interactions and strategic catalyst design to direct reactivity to the *meta*-position, bypassing the inherent electronic preference for *ortho-* and *para*-functionalization. Despite the progress, the field of *meta*-selective C–H functionalization is still in its early stages and further innovation is required to broaden its applicability and efficiency.

### 3.1. σ-bond activation strategy

A promising approach for achieving *meta*-C–H functionalization involved the use of ruthenium catalysts through arene σ-activation, particularly in substrates with azine directing groups.^126^ The key mechanistic feature of this approach was the formation of a ruthenium metallacycle intermediate, which preferentially facilitated radical addition at the position *para* to the Ru–C bond, thereby enabling selective functionalization at the *meta*-position. The initial developments by Frost^127^ and Ackermann^128^ demonstrated the versatility of Ru-catalyzed *meta*-functionalization across a variety of transformations, including sulfonylation, alkylation, halogenation, nitration, and carboxylation.^129–131^ However, these processes typically required elevated temperatures, which, while effective, limited the compatibility with sensitive functional groups and thus emphasized the necessity of developing methods that can be performed under milder reaction conditions.

**Scheme 16:**
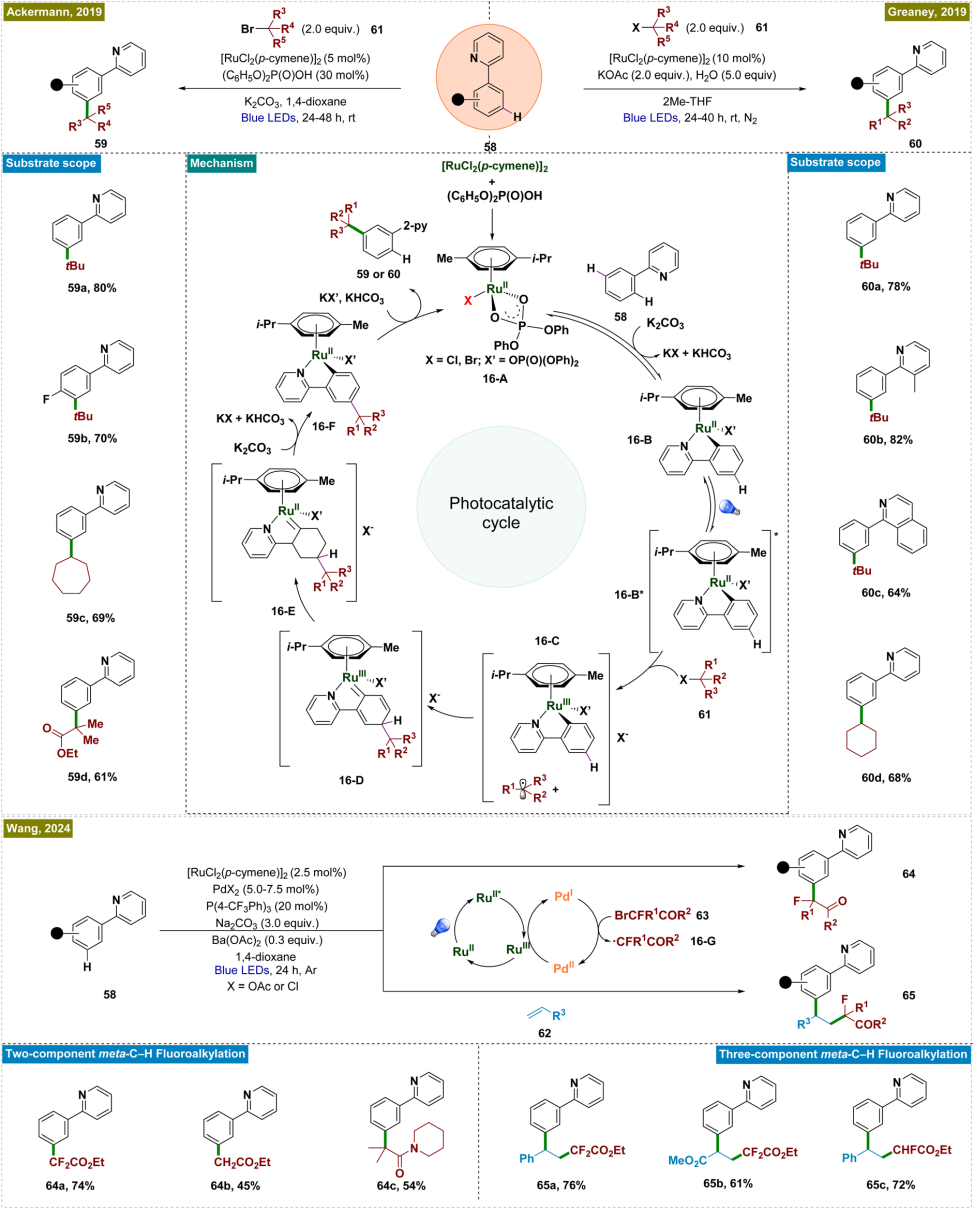
Photoinduced ruthenium-catalyzed *meta*-C–H alkylation with alkyl halides.

To address this limitation, a novel photochemical strategy was independently developed by the groups of Ackermann and Greaney in 2019, enabling Ru-catalyzed *meta*-C–H alkylation of 2-aryl pyridines under photocatalytic conditions at room temperature (Scheme 16).^132,133^ Both groups employed the [RuCl_2_(*p*-cymene)]_2_ catalyst, which formed a cyclometalated ruthenium complex (16-B) through reversible C–H ruthenation of 2-phenylpyridine. Upon irradiation of light, the Ru(ii) complex was photoexcited and acted as a single-electron donor, reducing the alkyl halide and forming an oxidized Ru(iii) complex (16-C). The resulting alkyl radical then underwent a *para*-selective addition, followed by intramolecular ligand-to-metal charge transfer (LMCT), re-aromatization, and protodemetalation, yielding the *meta*-functionalized product and regenerating the Ru-catalyst. Unlike in thermal catalysis, where the ruthenium metallacycle functioned as a nucleophile, the oxidized form under photochemical conditions served as a radical acceptor, driving distinct reactivity and expanding the reaction scope. This photochemical approach accommodated a broader range of functional groups, including tertiary and secondary unactivated alkyl bromides and synthetically valuable α-bromo esters.

Several mechanistic investigations were conducted by the groups of Ackermann^133^ and Greaney,^132^ including on/off experiments, cyclic voltammetry, and radical trapping, providing evidence that the photocatalytic reaction proceeded *via* single-electron transfer from the photoexcited ruthenium metallacycle, leading to the formation of alkyl radical intermediates. These investigations established the reaction's light dependency, with a relatively low quantum yield of 1.6%, and demonstrated that radical scavengers significantly inhibited the process. Cyclic voltammetry revealed a reversible oxidation event with an oxidation peak at 0.98 V in DCE, further supporting the proposed mechanism. Wang *et al.* recently extended this approach by developing a visible-light-mediated bimetallic catalysis system for *meta*-fluoroalkylation of arenes, utilizing a ruthenium–palladium dual catalyst strategy (Scheme 16).^134^ In this system, ruthenium served as both the directing group and the photoredox catalyst, while palladium was responsible for generating fluoroalkyl radicals. This methodology not only achieved high regioselectivity in alkylation but also exhibited broad functional group tolerance under mild reaction conditions. It is applicable to both two-component aromatic *meta*-fluoroalkylation reactions and three-component olefin difunctionalization, enabling the construction of complex molecular architectures. Mechanistic studies have clarified that ruthenium mediated both C–H activation and photoredox processes. Concurrently, the palladium cocatalyst facilitated the single-electron transfer (SET) process with bromofluoroalkanes (63), generating fluoroalkyl radicals (16-G) *via* Pd(0) or Pd(i) species. The distinct roles of ruthenium and palladium in this dual catalytic system worked efficiently to drive the catalytic cycle for *meta*-alkylation of arenes.

**Scheme 17:**
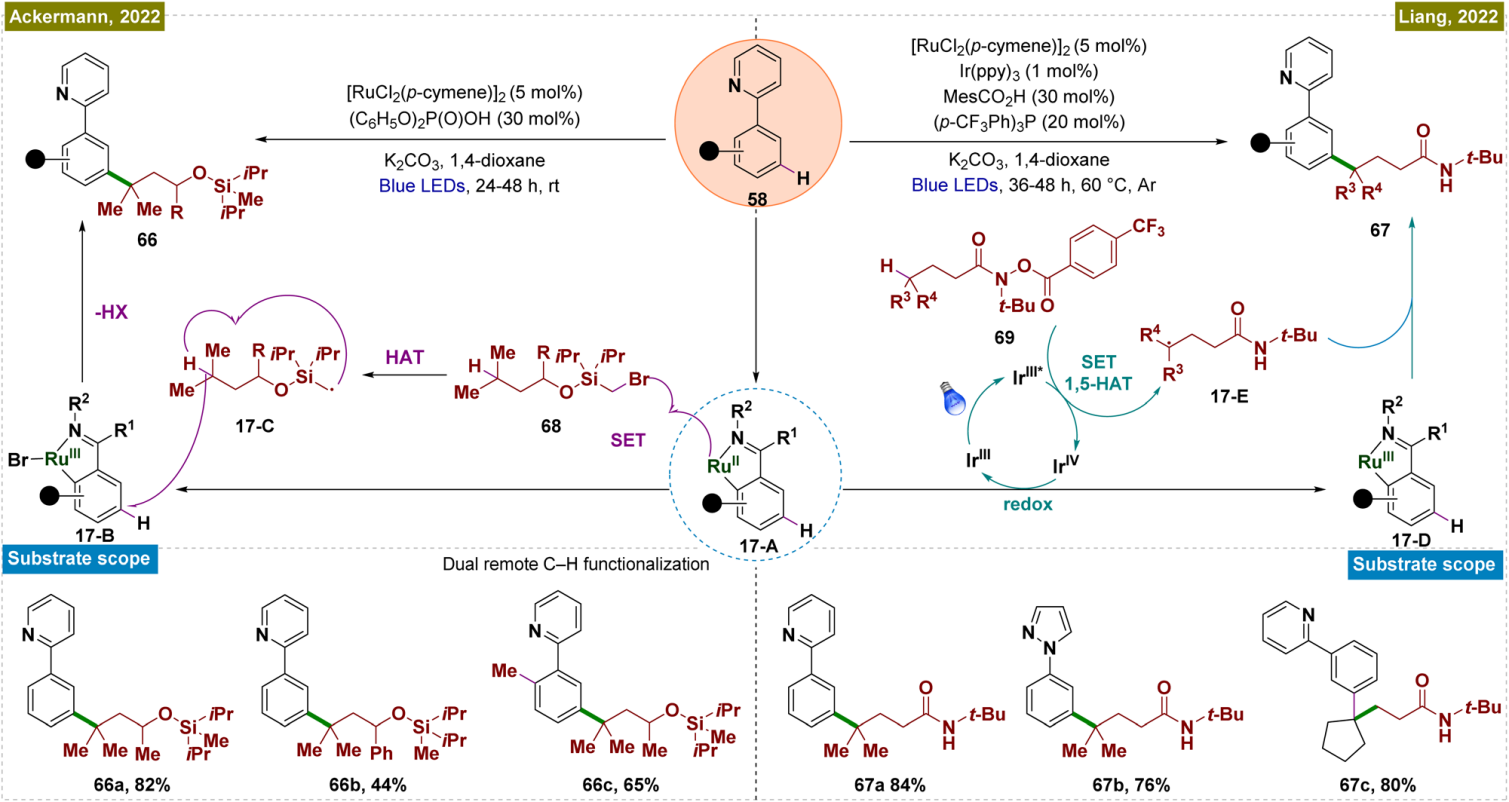
Site-selective coupling of remote C(sp^3^)–H/*meta*-C(sp^2^)–H bonds enabled by a Ru catalyst.

In general, *meta*-functionalization reactions have relied on substrates that generate radicals *via* SET with Ru catalysts, limiting the scope of *meta*-arene C–H bond functionalization. In the field of remote C(sp^3^)–H functionalization, the hydrogen atom transfer (HAT) process has emerged as a complementary and highly effective approach to the established cyclometallation and metal carbene pathways.^135^ This process is typically performed under mild reaction conditions, offering high efficiency and regioselectivity, with a preference for tertiary C–H sites over secondary and primary ones. In 2022, Liang's group introduced a novel strategy that integrated ruthenium-catalyzed *meta*-C–H functionalization with an aliphatic HAT process (Scheme 17).^136^ This method enabled the regioselective cross-dehydrogenative coupling of dual remote C–H bonds, specifically targeting inert γ-C(sp^3^)–H bonds in amides and *meta*-C(sp^2^)–H bonds in arenes, thereby facilitating the construction of *meta*-alkylated arenes. This new catalytic approach bypassed the reliance on SET with Ru catalysts, broadening the scope of *meta*-arene C–H bond functionalization reactions. In this process, Ir(ppy)_3_ acted as a photocatalyst, which upon irradiation, transitioned to its excited Ir(iii)* state. This state initiated a SET event to the hydroxamide substrate (69), causing N–O bond cleavage and forming a nitrogen-centered radical alongside Ir(iv). This radical underwent intramolecular 1,5-HAT, generating a carbon-centered radical (17-E). Simultaneously, the Ru(ii) complex (17-A), produced by C–H ruthenation of the arene, was oxidized by the Ir(iv) species, regenerating Ir(ppy)_3_ and forming a Ru(iii) complex. The carbon-centered radical (17-E) then selectively added to the C–H bond *para* to the Ru(iii) complex, finalizing the reaction. This process successfully functionalized primary, secondary, and tertiary C(sp^3^)–H bonds in amides, and it exhibited broad compatibility with a wide range of arenes, including natural products.

In the same year, Ackermann and co-workers reported transition metal-catalyzed double remote C(sp^2^)–H/C(sp^3^)–H functionalization *via* a radical relay strategy, employing a [RuX_2_(*p*-cymene)]_2_ catalyst (Scheme 17).^137^ In this innovative approach, the Ru catalyst simultaneously activated the *meta*-C(sp^2^)–H bond of arenes and the remote C(sp^3^)–H bond of alkanes in a single operation, eliminating the necessity of an additional photocatalyst. The mechanism began with the formation of a Ru(ii) metallacycle complex (17-A), followed by a SET between the metallacycle and the alkyl substrate (68), generating a Ru(iii) intermediate (17-B) and a silyl methyl radical (17-C). This radical subsequently underwent a HAT process to form a more stable carbon-centered radical, which then added to the *para*-position relative to the C–Ru bond. Mechanistic studies further revealed that continuous irradiation of light was not required throughout the entire reaction but was essential in the initial phase to activate the [RuX_2_(*p*-cymene)]_2_ precatalyst, forming the catalytically active Ru(ii) complex. The general applicability of this method was demonstrated through gram-scale synthesis, confirming its scalability. Additionally, the combination of dual remote C–H functionalization with Ru-catalyzed *ortho*-C–H functionalization was shown to be feasible in a one-pot process, underscoring the versatility and practicality of this approach.

### 3.2. Template assisted *meta*-C–H bond functionalization

**Scheme 18:**
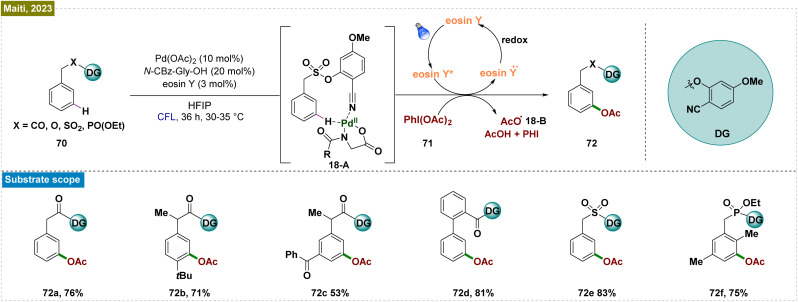
Photoinduced template mediated Pd-catalyzed *meta*-oxygenation of arenes.

Despite significant advancements in visible-light-mediated Ru-catalyzed *meta*-functionalization, these transformations consistently depended on the formation of a ruthenium metallacycle intermediate, which constrained the reaction scope. A potential solution involved direct releasing of the reactive metal catalyst near the targeted remote C–H bond. However, this strategy required the formation of a larger macrocyclic pretransition state, resulting in a thermodynamically unfavorable cyclometalated intermediate. To overcome these obstacles, Yu and co-workers introduced an end-on template with a linear nitrile functionality as the directing group.^138^ This design employed a long tethering fragment and a rigid, linear nitrile that effectively positioned the Pd-catalyst close to the *meta*-C–H bond, thereby minimizing the competing *ortho*-palladation, which was typically associated with significant ring strain due to the small cyclic pretransition state. Building on this concept and their prior success with nitrile-based templates for *meta*-C–H functionalization, Maiti *et al.* developed an efficient and selective photoinduced *meta*-oxygenation of substrates such as phenylacetic acids, biaryl acids, and alcohols through the synergistic combination of photoredox and Pd catalysis (Scheme 18).^139^ Unlike thermal catalysis, which demands elevated temperatures and excess acetylating agents for optimal selectivity, this catalytic system operated under mild conditions. The mechanism initiated with the weak Pd–nitrile interaction, positioning the Pd–ligand complex near the *meta*-C–H bond. Subsequently, photoexcitation activated the C–H bond, forming a palladacycle intermediate (18-A). The eosin Y-assisted reduction of PhI(OAc)_2_ (71) generated an acetoxy radical (18-B) that coordinated with this intermediate, leading to a Pd(iii) complex. Subsequent oxidation by eosin Y produced a Pd(iv) intermediate, which underwent reductive elimination to yield the *meta*-acetoxylated product (72) and regenerated the Pd(ii) catalyst. Radical quenching experiments and EPR analysis confirmed the radical nature of the process.

**Scheme 19:**
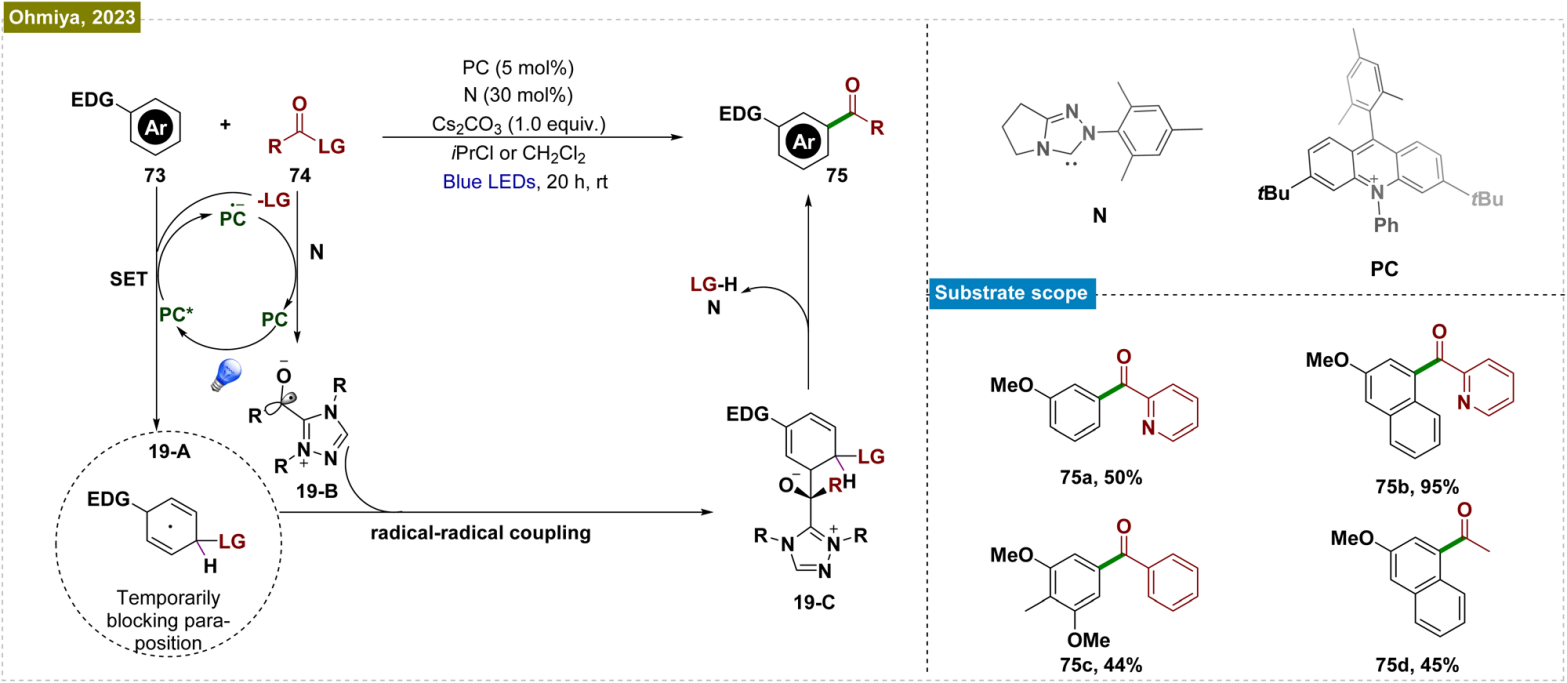
NHC and organic photoredox-catalyzed *meta*-selective acylation of electron-rich arenes without directing group assistance.

The use of directing groups is a widely adopted and critical strategy for achieving *meta*-selective functionalization of C–H bonds in arenes. However, the requirement for prefunctionalization and postfunctionalization steps restricted the substrate scope and rendered these processes step intensive. To overcome this limitation, Ohmiya *et al.* developed a method combining N-heterocyclic carbene (NHC) and organic photoredox catalysis for *meta*-selective acylation of electron-rich arenes, eliminating the need for directing groups (Scheme 19).^140^ The process began with SET oxidation of the arene (73), forming a radical cation. Simultaneously, the NHC catalyst (N) reacted with acyl imidazole (74) to generate an acyl azolium intermediate and an imidazolide anion (LG). This anion acted as a nucleophile, creating a cyclohexadienyl radical (19-A) that enabled *meta*-selective acylation. Subsequent SET reduction of acyl azolium gave the ketyl radical (19-B) and closed the photoredox cycle. The reaction was completed by radical–radical coupling of the cyclohexadienyl radical with the ketyl radical and rearomatization. This catalytic system efficiently functionalized a variety of mono- and disubstituted electron-rich arenes, including acenes, using a broad range of carboxylic acid derivatives.

## 4. *Para*-selective C–H functionalization

*Para*-selective C–H functionalization of aromatic compounds is a challenging task due to the difficulty of targeting specific C–H bonds in the symmetrical and reactive nature of the aromatic ring.^6,141^ The *ortho*- and *meta*-positions typically exhibit greater reactivity, making it hard to selectively functionalize the *para*-position. This challenge arises from a combination of electronic and steric effects, as the *para*-position is often less activated and harder to distinguish from other positions on the ring.^142^ However, recent advances in photoredox catalysis offer new strategies to address this issue. Photoredox catalysis uses light-activated catalysts to trigger selective radical or electrophilic processes, providing a gentler and more precise approach to C–H functionalization.^143^ By carefully designing photocatalysts, researchers can exploit their unique electronic properties to promote *para*-selectivity.^143^ This is achieved through catalyst-driven control over reaction mechanisms, which modulate reactivity and regioselectivity by stabilizing key intermediates.^143,144^ In addition, photoredox catalysis enables the use of mild oxidants and reductants, improving selectivity and tolerance to different functional groups compared to conventional methods.^144^ The tunability of light as an energy source allows for fine control over reaction conditions, resulting in enhanced selectivity for *para*-position functionalization in aromatic systems.^143^

### 4.1. Radical-cation pathway

In recent decades, organometallic cross-coupling has become a cornerstone for constructing intricate aromatic compounds from pre-functionalized precursors.^145^ Photoredox catalysis offers a direct and efficient approach to arene functionalization, bypassing the necessity of pre-functionalization.^23,143,144,146^ One of the earliest strategies developed for the regioselective functionalization of phenyl rings involves the oxidation of aromatic compounds, followed by nucleophilic attack by the participating reagent to functionalize the phenyl ring. Fukuzumi is recognized as one of the pioneers in photocatalytic aromatic ring functionalization using this approach. High regioselectivity is typically attributed to: (a) specific interactions between the catalyst and substrate or reagent and substrate, (b) the electronic properties of the substrate, (c) the steric characteristics of the substrate or catalyst, or (d) particular mechanistic pathways.^86,147–153^

**Scheme 20:**
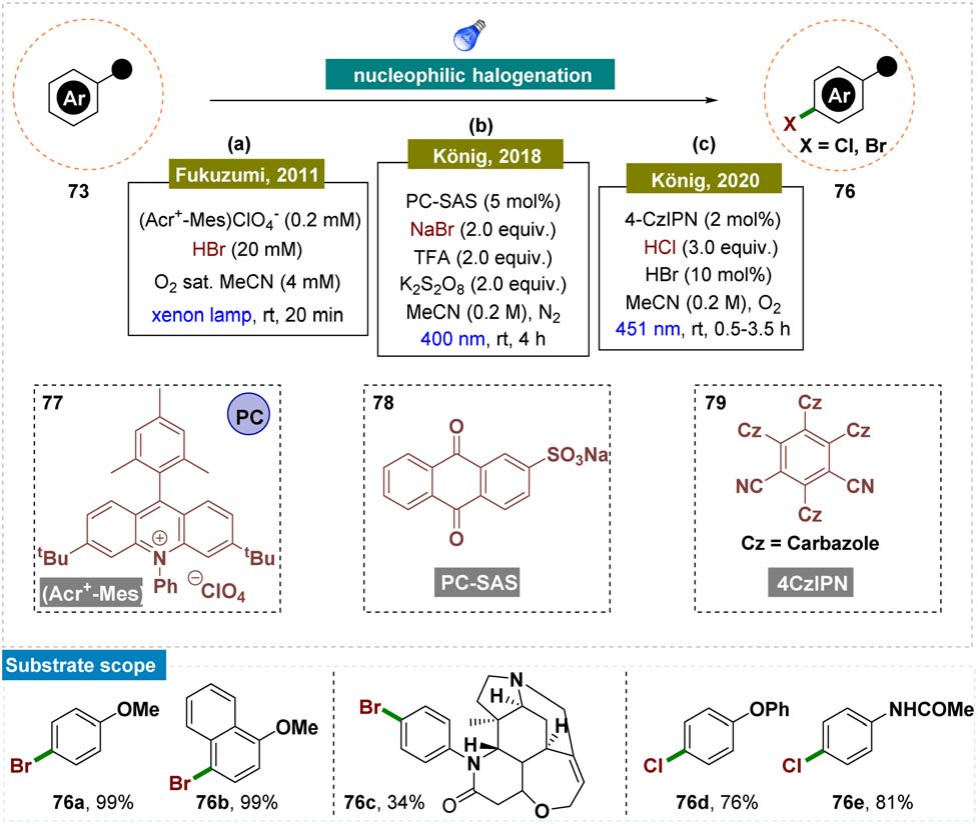
*Para*-selective C–H bond halogenation of arenes.

In 2011, Fukuzumi and team disclosed photocatalytic bromination of aromatic hydrocarbons by using molecular oxygen and hydrogen bromide, which proceeded efficiently, yielding selectively monobrominated products with 9-mesityl-10-methylacridinium ion (Acr^+^-Mes) 77 as a photocatalyst under the irradiation of visible light (Scheme 20a).^147^ The photocatalytic turnover number reached 900, based on the initial concentration of Acr^+^-Mes. Reactive radical intermediates involved in the photocatalytic cycle were successfully detected by using laser flash photolysis. The reaction mechanism began with photoinduced electron transfer from the mesitylene moiety to the singlet excited state of the acridinium ion, forming the electron-transfer state (Acr˙-Mes˙^+^). This was followed by electron transfer from aromatic hydrocarbons to the mesitylene radical cation and from the acridinyl radical to O_2_. The radical cations of the aromatic hydrocarbons then reacted with Br^−^, selectively producing the monobrominated products.

However, the reaction conditions developed by Fukuzumi were harsh due to the use of corrosive HBr and a high-power xenon lamp, which limited the substrate scope. To overcome this, in 2018, König and colleagues utilized nucleophilic addition to an aromatic radical-cation intermediate for a bromination reaction under the irradiation of visible-light (Scheme 20b).^151^ In their study, they developed reaction conditions where the oxidation potential of the sodium anthraquinone-2-sulfonate (SAS) photocatalyst 78 increased from 1.8 V to approximately 2.3 V *vs.* SCE upon the addition of a Brønsted acid. This enhanced photooxidation ability of protonated anthraquinone was then applied for the regio-selective oxidative bromination of electron-rich (hetero)arenes and drugs, yielding good results. The reaction was carried out under mild conditions, which were compatible with various functional groups, including double and triple bonds, ketones, amides, amines, hydroxyl groups, carboxylic acids, and carbamates.

Later in 2020, the same group described a methodology where electron-rich arenes were oxidatively photochlorinated using catalytic amounts of bromide ions, visible light, and 4CzIPN 79 as an organic photoredox catalyst (Scheme 20c).^153^ From their previous report, they have successfully replaced the stoichiometric oxidant with new and efficient catalytic conditions. In this process, the substrates were first brominated *in situ via* a photoredox-catalyzed oxidation, followed by a photocatalyzed *ipso*-chlorination, resulting in the desired compounds with high *ortho*/*para*-regioselectivity. Dioxygen acted as a green and efficient terminal oxidant, while the use of aqueous hydrochloric acid as the chloride source minimized the formation of saline by-products.

**Scheme 21:**
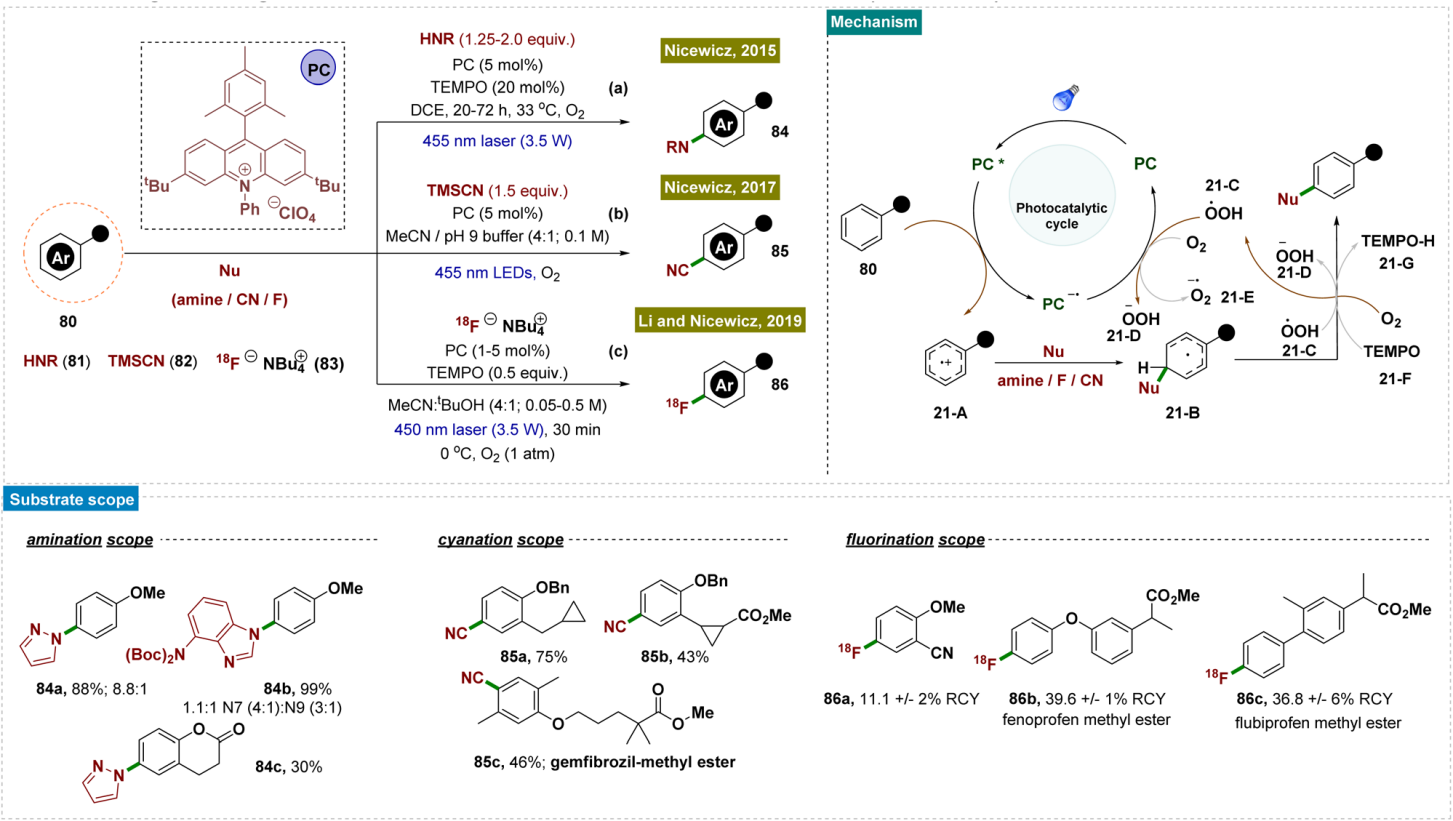
*Para*-selective C–H bond functionalization of arenes.

Moving forward, Nicewicz and co-workers developed several photocatalytic strategies to functionalize the arene C–H bond for the formation of C–N,^148^ C–C and C–F^150,152^ bonds by applying this radical-cation pathway (Scheme 21). In all of these cases, the high regioselectivity was attributed to the unique catalytic cycle and the involvement of an electron-rich arene system.

In 2015, they developed a photoredox catalyst system comprising an acridinium photooxidant and a nitroxyl radical 21-F that enabled site-selective amination of diverse aromatics with heteroaromatic azoles (as nucleophiles), compounds of significant pharmaceutical interest (Scheme 21a).^148^ Furthermore, this approach utilized ammonia directly, providing an atom-economical route to anilines without requiring pre-functionalization of the aromatic substrate.

In 2019, a heterogeneous version of photoredox catalysts was designed by the Wang and König groups for the formation of C–N bonds regioselectively with broad tolerance of substrates and high efficiency, by employing molecular oxygen as the terminal oxidant.^154^ This semiconductor-based photoredox system enabled C–H amination without the necessity of metals, ligands, strong oxidants, or additives, and was recyclable up to 6 cycles, offering a sustainable and efficient approach to C–H functionalization with broad synthetic utility. Mechanistic studies, supported by electron spin resonance (ESR) data, KI-starch tests, and control experiments, identified superoxide anion radicals (O_2_˙^−^) and H_2_O_2_ as the reactive oxygen species.

In 2017, as follow-up work, Nicewicz and team reported a novel method for synthesizing aromatic nitriles through direct C–H functionalization by using an acridinium photoredox catalyst and trimethylsilyl cyanide 82 (as a nucleophile) under ambient conditions (Scheme 21b).^150^ This reaction was performed at room temperature and tolerated a wide range of functional groups, including electron-donating and -withdrawing groups, halogens, nitrogen- and oxygen-containing heterocycles, and even aromatic moieties found in pharmaceutical compounds.

Two years later, in 2019, they disclosed a mild process for direct [18F] fluorination of aromatic C–H bonds using an [18F] fluoride salt 83 under photocatalytic conditions. This photoredox-catalyzed approach enabled the synthesis of a diverse range of [18F]-labeled arenes and heteroaromatics, including those relevant to pharmaceutical applications (Scheme 21c).^152^ These radiolabelled compounds held promise as diagnostic agents and provided valuable insights into the *in vivo* behaviour of their unlabelled counterparts, as demonstrated in preliminary animal studies. In this context, it is important to note that positron emission tomography (PET) plays a crucial role in drug discovery, development, and medical imaging. However, the lack of efficient and straightforward radiolabeling methods for aromatic C–H bonds presents a significant challenge, hindering progress in PET radiotracer development.

Mechanistically, all these reports followed similar reaction pathways as described in Scheme 21. According to the authors, the mechanism of this reaction was not fully understood and is currently under investigation. They proposed that TEMPO facilitated the aromatization of radical intermediate 21-B through direct H-atom abstraction (Scheme 21). The generation of radical intermediate 21-B was achieved through the trapping of radical-cation species 21-A with a suitable nucleophile (81/82/83). Alternatively, radical 21-B was trapped by O_2_, forming a 1,3-cyclohexadienyl peroxyl radical, which underwent internal elimination to yield products 84–86 and the hydroperoxyl radical 
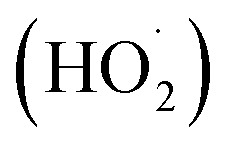
. As suggested in the literature, O_2_ oxidized the acridine radical Mes-Acr˙ (Mes, mesityl; Acr, acridinium), regenerating acridinium (Mes-Acr^+^) and superoxide (O_2_˙^−^), although other intermediates, such as HOO˙, might also contribute to catalyst turnover. The highly basic superoxide likely deprotonated intermediate 21-B, followed by hydrogen atom transfer with TEMPO-H, ultimately forming H_2_O_2_ and regenerating TEMPO. The observed reduction in unwanted byproducts when TEMPO was present supported the proposed role of TEMPO-H in scavenging reactive oxygen-centered radicals like 
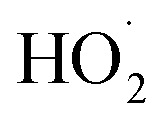
. Although the half-wave redox potential of TEMPO [*E*_1_/_2_ (TEMPO˙/TEMPO^+^) = +0.62 V *vs.* Ag/AgCl] suggested possible oxidation by cat^+^*, the use of 20 mol% TEMPOnium-BF_4_ yielded similar results to TEMPO in the aryl amination reaction, indicating a common mechanistic intermediate, presumably TEMPO, generated through electron transfer from cat˙ [*E*_1_/_2_ (cat^+^/cat˙) = −0.47 to −0.58 V *vs.* SCE] to TEMPOnium. In the absence of cat^+^, no aryl amine 84 was formed with 20 mol% TEMPO, though trace product formation was observed with 20 mol% TEMPOnium-BF_4_ when the acridinium photocatalyst was omitted.

The direct incorporation of a trifluoromethyl group onto an aromatic ring *via* a radical pathway has been widely studied,^155^ but achieving highly *para*-selective C–H trifluoromethylation of specific arenes has remained challenging.

**Scheme 22:**
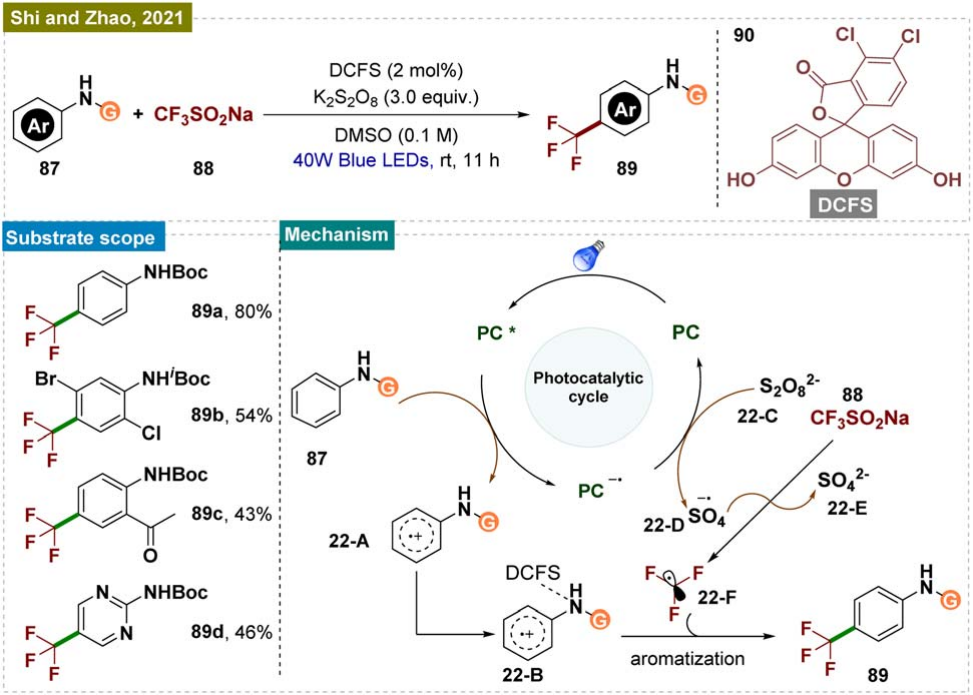
*Para*-selective C–H bond trifluoromethylation of arenes.

In 2021, Shi, Zhao, and their team reported a light-promoted, 4,5-dichlorofluorescein (DCFS, 90)-enabled *para*-selective C–H trifluoromethylation of arylcarbamates using the Langlois reagent 88 (Scheme 22).^86^ Preliminary mechanistic studies suggested that the activated organic photocatalyst, when coordinated with the arylcarbamate, facilitated the *para*-selective trifluoromethylation. The method's synthetic significance was underscored by its successful application on a ten-gram scale.

Mechanistically (Scheme 22), a trifluoromethyl radical 22-F was generated through a known process using 4,5-DCFS 90 as an organic photocatalyst and K_2_S_2_O_8_22-C as the oxidant. The oxidation of DCFS formed the key intermediate 22-A*via* a single-electron transfer (SET) process, which then underwent tautomerization to produce intermediate 22-B with the activated DCFS. The steric bulk of DCFS increased the hindrance at the *ortho-* and *meta*-positions on the phenyl ring, promoting highly *para*-selective trifluoromethylation. However, the possibility that the free trifluoromethyl radical 22-F reacted directly with the DCFS-activated phenyl ring could not be completely ruled out.

**Scheme 23:**
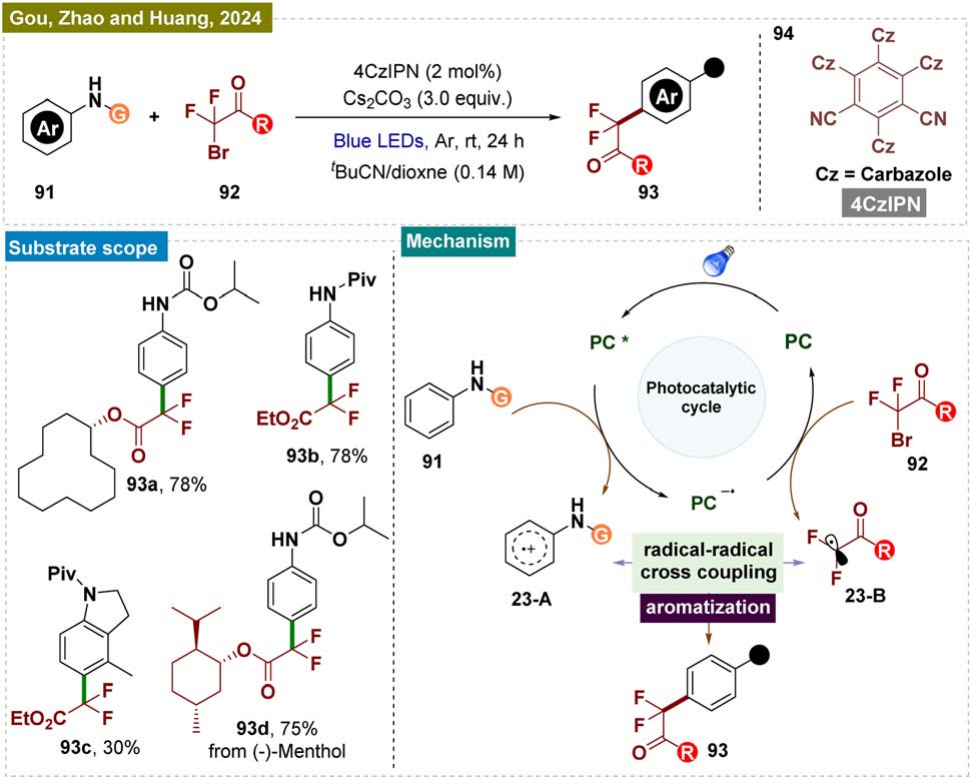
*Para*-selective C–H difluoromethylation of arenes.

Apart from trifluoromethylation of secondary amines, synthesis of other fluorinated compounds, such as difluoromethylated aromatics, is also in high demand due to their synthetic and biological value.^156^ Recently in 2024, Guo, Zhao, Huang, and colleagues introduced a green and environmentally friendly modular platform for the sustainable *para*-difluoromethylation of readily available aromatic amines 95 using a metal-free photocatalytic system (Scheme 23).^157^ This reaction, for the first time, exhibited excellent functional group tolerance and a broad substrate scope, employing a radical–radical coupling C–H functionalization strategy to efficiently generate a range of valuable difluoromethylated products 97. Comparative experiments revealed that the activating group is crucial for electronic control of regioselectivity and for preventing the decomposition of arylamines. Moreover, the high atom economy achieved in the derivatization of complex molecules underscored the synthetic utility and practicality of this approach.

A plausible reaction mechanism was proposed by the authors and is illustrated in Scheme 23. Compound 95 underwent reductive quenching by the excited state 4CzIPN*, generated upon visible light irradiation, leading to the formation of 4CzIPN˙^−^ and a key transient arene radical-cation 23-A (supported by Stern–Volmer emission quenching studies). Subsequently, the reduced photocatalyst 4CzIPN˙^−^ transferred a single electron to alkyl bromide 96, generating the difluoroalkyl radical 23-B and restoring the photocatalyst 4CzIPN. The sp^3^–sp^2^ coupling between the ˙CF_2_CO_2_Et radical 23-B and arene cation radical 23-A then produced a cationic intermediate, which underwent deprotonation by a base to yield product 97.

### 4.2. Radical trifluoromethylation

**Scheme 24:**
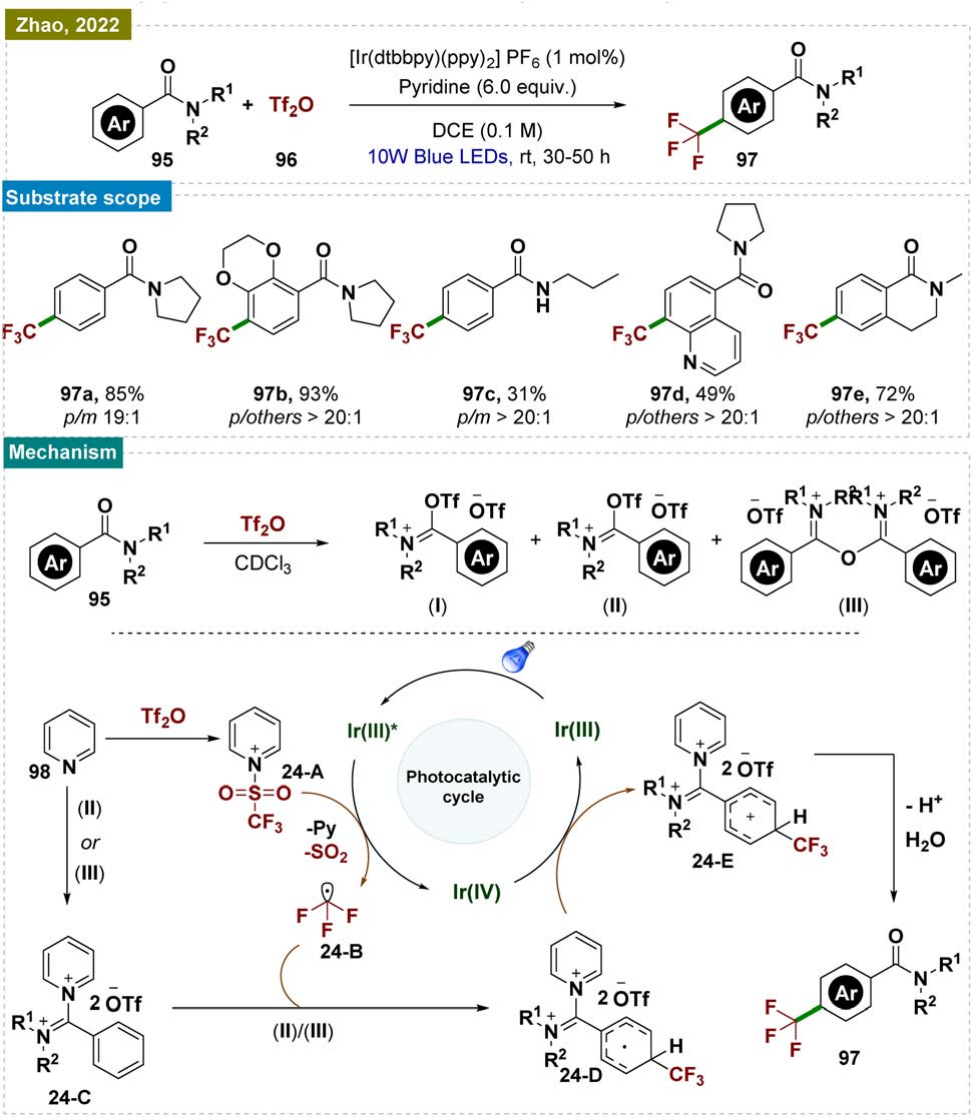
*Para*-selective C–H bond trifluoromethylation of benzamide.

Understanding the importance of aromatic trifluoromethylation^158^ regioselectively, Zhao and team presented a novel strategy for *para*-selective C–H trifluoromethylation of benzamide derivatives 91 through iminium activation (Scheme 24).^159^ This approach facilitated a radical nucleophilic substitution rather than a radical electrophilic substitution, driven by iminium activation, which lowered the lowest unoccupied molecular orbital (LUMO). This method was compatible with a wide range of substrates, producing predominantly *para*-trifluoromethylated products with high selectivity.

A plausible reaction mechanism was proposed (Scheme 24). Initially, pyridinium complex 24-A and iminium intermediate 24-C are formed *in situ*.^160^ Upon irradiation with blue LEDs, the excited-state Ir^III^* was generated, which reduced complex 24-A to radical species 24-B through a single electron transfer (SET) process.^160^ The –CF_3_ radical 24-B, released from radical species 24-A, then underwent nucleophilic addition to iminium intermediate 24-C, forming intermediate 24-D. The selective activation at the *para*-position of the iminium salt resulted in high *para*-selectivity, avoiding *meta*-selectivity.^149,161^ Another SET event between radical 24-D and Ir^IV^ regenerated Ir^III^, continuing the catalytic cycle and leading to intermediate 24-E. Deprotonation of intermediate 24-E followed by hydrolysis of iminium intermediate 24-E yields the desired product 93.

However, a radical chain mechanism cannot be ruled out. Intermediate 24-D underwent deprotonation to form a radical anion, which directly reduced complex 24-A to radical species 24-B, initiating a radical chain process. While –CF_3_ radicals kinetically favored addition at the *para*-position, the exact direction of electron transfer in the transition state remained unconfirmed, requiring further investigation. Another plausible explanation was that cyclohexadienyl radical 24-D was thermodynamically stabilized by delocalization with the iminium group, favoring *para*-trifluoromethylation. Given the broad applicability of this protocol to various functionalized substrates, it was also likely that radical 24-D was both kinetically preferred and thermodynamically more stable.

### 4.3. Functionalization of unprotected functional groups bearing a phenyl ring for C–C bond formation

**Scheme 25:**
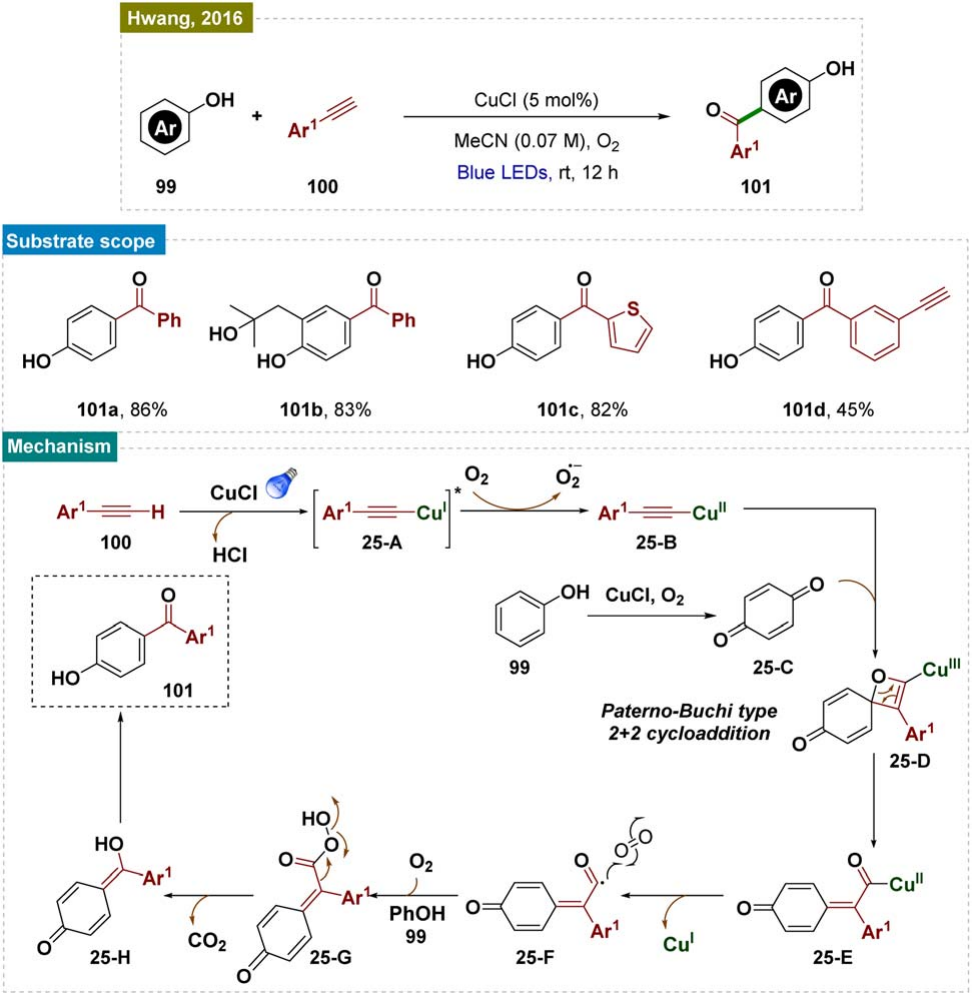
*Para*-selective C–H bond functionalization of unprotected phenols.

Aromatic substrates with unprotected functional groups present significant challenges due to their delicate nature. Free groups such as –OH and –NH_2_ often participate in side reactions, making selective functionalization difficult.^162^ Achieving regioselective functionalization of these substrates typically requires specialized catalytic conditions. In this context, Hwang and colleagues have developed a visible-light-mediated, copper-catalyzed photoredox process that enables direct oxidative coupling of phenols 99 with terminal alkynes 100 at room temperature (Scheme 25).^163^ This method facilitated *para*-selective formation of hydroxyl-functionalized aryl and alkyl ketones through the cleavage of the C≡C triple bond.

Visible-light irradiation of the *in situ* generated Cu(i)-phenylacetylide produced a long-lived excited state of Cu(i)-phenylacetylide 25-A. This excited state underwent a single-electron transfer (SET) process, donating an electron to O_2_ and forming the intermediate Cu(ii)-phenylacetylide 25-B and a superoxide radical anion, as confirmed by EPR experiments. Given the redox potential of 25-A (−2.048 V *vs.* SCE in CH_3_CN) and the strong electron affinity of O_2_, it was likely that this SET process readily occurred between the photoexcited triplet Cu(i)-phenylacetylide and O_2_. Simultaneously, phenol 99 was oxidatively converted to benzoquinone (BQ) 25-C by the Cu(ii)-superoxo intermediate. Next, the Paternò–Büchi-type [2 + 2] cycloaddition between Cu(ii)-phenylacetylide 25-B and benzoquinone (BQ, 26-C) formed a labile Cu(ii)-oxetene intermediate 26-D. This oxetene ring underwent rearrangement to produce the Cu(ii)-coordinated quinone methide 26-E, which rapidly fragmented to generate an acyl radical intermediate 26-F and Cu(i). Subsequent dark reactions with molecular oxygen (O_2_) and the abstraction of a phenolic proton resulted in the formation of peracid 26-G. This peracid then underwent radical cleavage, releasing CO_2_ and producing the hydroxyl-substituted quinone methide 26-H. Finally, keto–enol tautomerism (*via* aromatization) led to the formation of the aryl ketone (101).

**Scheme 26:**
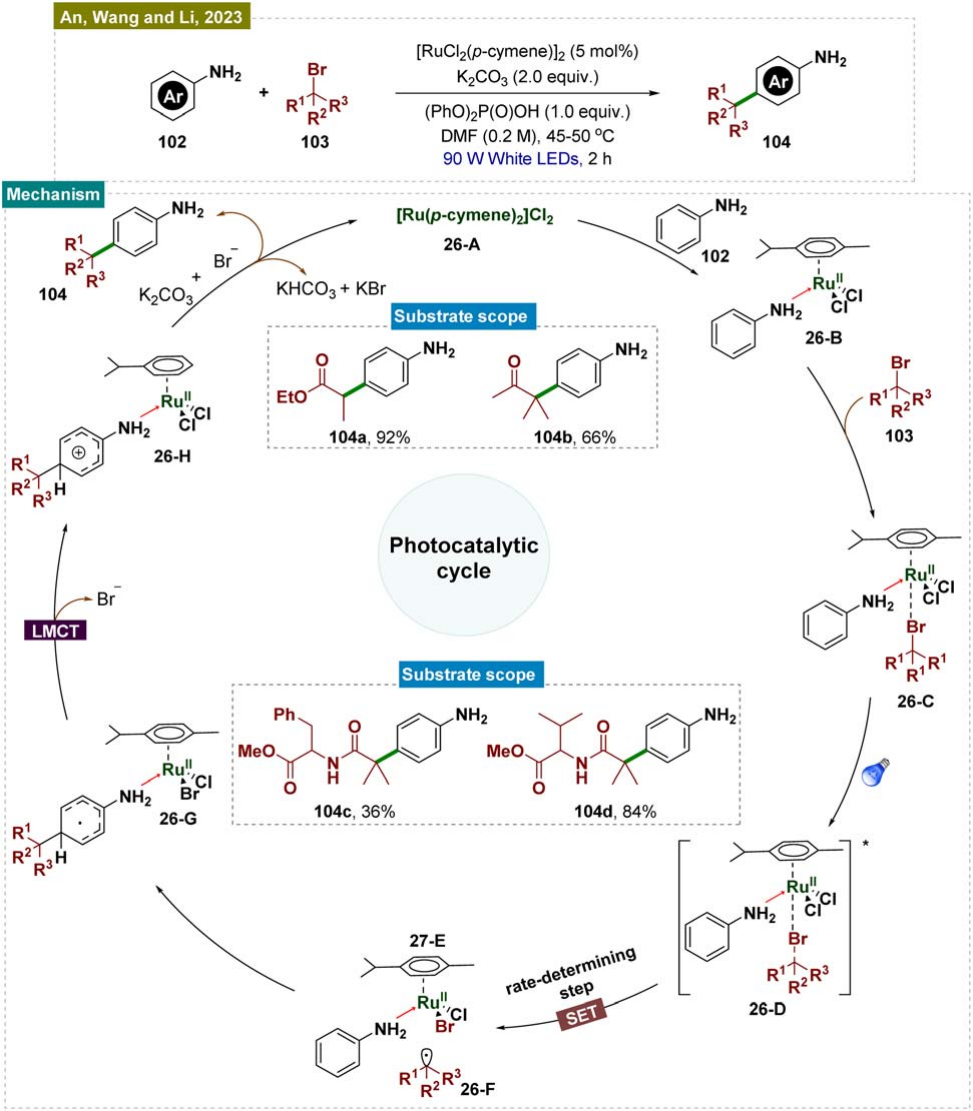
*Para*-selective C–H bond alkylation of unprotected anilines.

In addition to unprotected phenols, free anilines are also key structural motifs in organic and pharmaceutical chemistry.^164^ Therefore, functionalizing anilines while preserving the integrity of the –NH_2_ group is a challenging task, requiring selective reaction conditions to avoid undesired modifications. In 2023, An, Wang, and Li reported a visible light-mediated method for *para*-alkylation of unprotected anilines 102 using a Ru(ii)–aniline complex, achieving remarkable efficiency in just 2 hours under mild conditions (Scheme 26).^165^ This protocol demonstrated excellent functional group tolerance, enabling late-stage functionalization of natural products and pharmaceuticals. Mechanistic studies indicated that the unique Ru(ii)–aniline complex played a crucial role in both initiating the reaction and ensuring *para*-selectivity.

Mechanistically (Scheme 26), [RuCl_2_(*p*-cymene)]_2_ first coordinated with aniline 102 to form complex 26-B, which then reacted with alkyl halide 103 to produce intermediate 26-C. Upon exposure to visible light, 26-C was excited to its active state, 26-D. This excited state underwent single-electron transfer (SET) to yield Ru(iii) species 26-E and an alkyl radical 26-F. The alkyl radical 26-F attacked the aromatic ring at the position *para* to the ruthenium, forming intermediate 26-G. Subsequently, a ligand-to-metal charge transfer (LMCT) occurred, generating complex 26-H. Finally, 26-H underwent rearomatization, released the *para*-alkylated product 104 and regenerated the ruthenium(ii) species [RuCl_2_(*p*-cymene)]_2_.

**Scheme 27:**
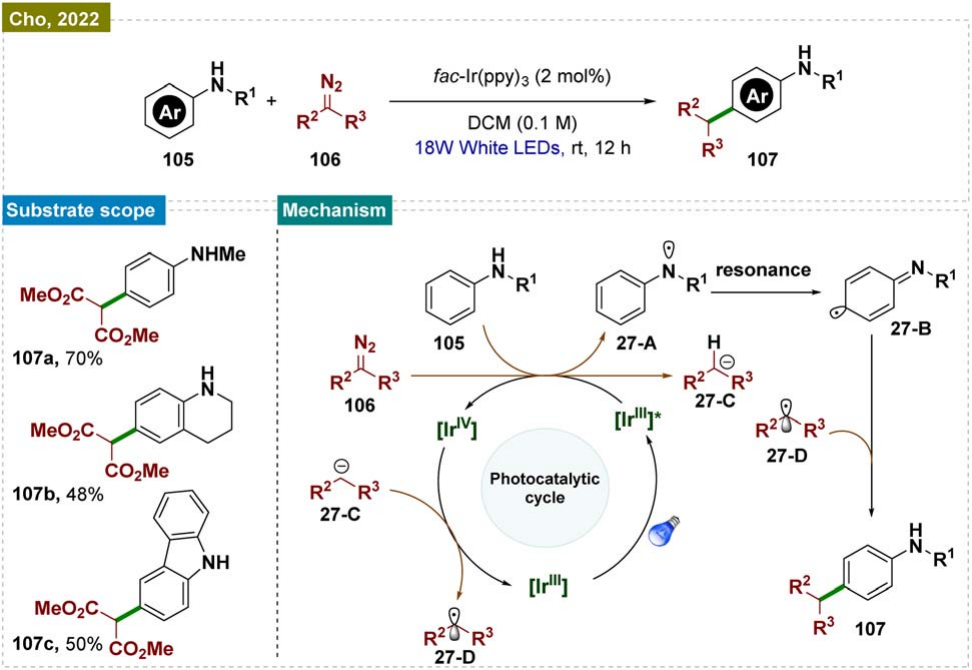
*Para*-selective C–H bond alkylation of arenes.

In 2022, the Cho group developed a visible-light-induced method for *para*-selective C–H functionalization of anilines 105, avoiding N–H insertion, using diazomalonates 106 in the presence of an Ir(iii) photocatalyst (Scheme 27).^166^ The *para*-selective radical–radical cross-coupling occurred *via* C-centered radical intermediates generated from both anilines 105 and diazomalonates 106. The reaction pathway for this selective C–C coupling was confirmed through electrochemical and photophysical experiments and computational studies.

Mechanistically (Scheme 27), upon visible-light irradiation, *fac*-Ir(ppy)_3_ underwent photoexcitation, resulting in a metal-to-ligand charge-transfer excited state [Ir^IV^]* (E*(Ir^IV^/*Ir^III^) = −1.58 V in DCM). This excited state was oxidatively quenched *via* single-electron transfer to 106 (*E*_red_, −1.40 V *vs.* SCE), producing [Ir^IV^] and the radical anion [106]˙^−^. Subsequent hydrogen atom abstraction (HAT) from 105 by [106]˙^−^ generated intermediate 27-C, releasing N_2_ and forming the persistent radical intermediate 27-A, which was stabilized by resonance across the phenyl ring. Meanwhile, 27-C donated an electron to [Ir^IV^], regenerating the ground state [Ir^III^] and creating the key C-centered radical species 27-D. The reaction was concluded with radical–radical coupling between 27-D·and 27-B, followed by rearomatization, yielding the *para*-C–C coupled product 107.

### 4.4. Dual-photocatalytic pathway

In addition to photoredox catalysis, the development of dual catalytic protocols further broadens the scope of reactivity available in organic synthesis.^167^ Dual-photocatalysis can be classified into two types:^168^ (a) the combination of photoredox and organocatalysis for mainly asymmetric transformations, and (b) metallaphotoredox, which enables access to odd-electron reactivity in transition metal catalysis, both involving radical species.

**Scheme 28:**
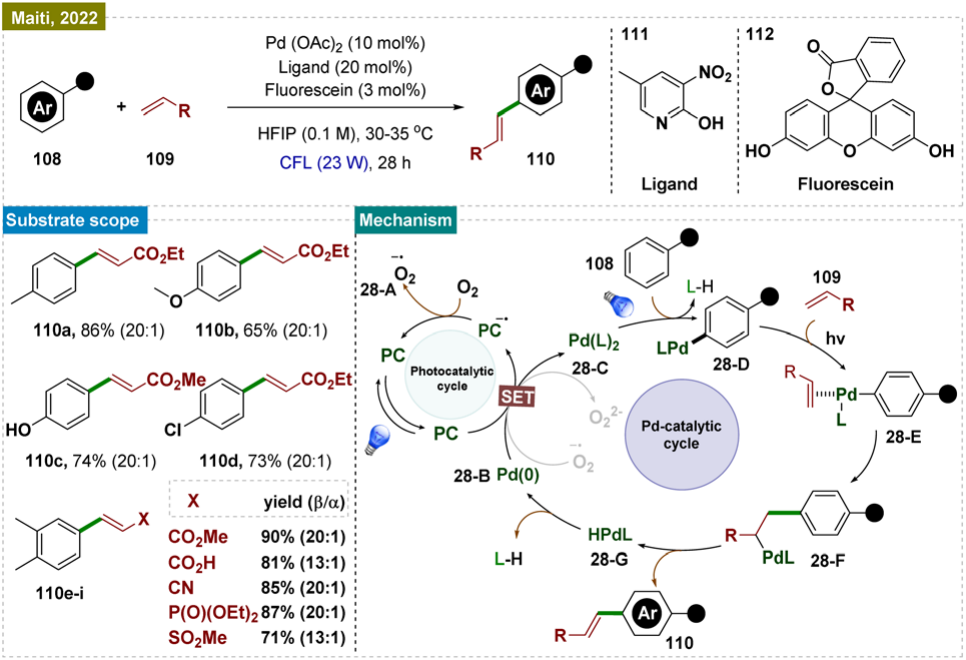
*Para*-selective C–H bond alkenylation of arenes.

In 2022, Maiti and colleagues developed a photoredox catalytic system combining palladium with an organo-photocatalyst (PC), enabling oxidative olefination with high regioselectivity across a broad range of arenes and heteroarenes (108) (Scheme 28).^169^ This system utilized visible light to conduct “regioresolved” Fujiwara–Moritani reactions without the need for silver salts or heat. The catalyst was versatile, accommodating both proximal and distal olefination through the use of specific directing groups (DGs), making it effective for C(sp^2^)–H olefination across a wide spectrum. The transformation underscored the dual function of visible light as both an oxidant and an activator, facilitating: (a) highly regioselective olefination of (hetero)arenes *via* a nondirected approach; (b) streamlined synthesis of phenolic natural products; (c) photoinduced atroposelective generation of axial chirality using a chiral ligand; (d) the first distal functionalization *via* photocatalysis; and (e) late-stage functionalization of biologically relevant molecules. Several control experiments were conducted by the authors to confirm key mechanistic aspects. To investigate whether a radical pathway was responsible for the formation of the dimerization product, the CDC reaction was performed in the presence of radical scavengers, as well as through a radical clock experiment using vinyl cyclopropane. In both cases, the yields remained unaffected, indicating a non-radical pathway. Additionally, the authors demonstrated that the C–H activation step was facilitated by the Pd catalyst and visible light, with the photocatalyst (PC) playing no significant role in this process. Based on the mechanistic investigations, a plausible mechanistic proposal was made (Scheme 28), suggesting that the direct excitation of the *in situ* generated Pd complex drove the C–H activation step. In line with previous studies, it was proposed that excitation of the photocatalyst allowed for the mild reoxidation of Pd(0) 28-B to Pd(ii) 28-A in the presence of oxygen (Scheme 28). As an alternative to the proposed oxidation step, it was also possible that the regeneration of the photocatalyst from its reduced state led to the reduction of molecular oxygen (O_2_), forming [O_2_]˙^−^28-A. In the oxidation step involving Pd(0) 28-B to Pd(ii) 28-A, O_2_ similarly participated in a single electron reduction process, generating [O_2_]˙^−^. This radical anion species, being highly short-lived, then rapidly underwent a protonation. The generated Pd(ii) complex underwent a C–H insertion reaction at the *para*-position of the arene, forming intermediate 28-D. This intermediate then reacted with olefin substrates to produce species 28-E, followed by the formation of species 28-F. Subsequently, dehydrogenative elimination from 28-F led to the formation of product 110 and the intermediate LPd-H (28-G), which readily converted to Pd(0) (28-B) through the elimination of L-H.

**Scheme 29:**
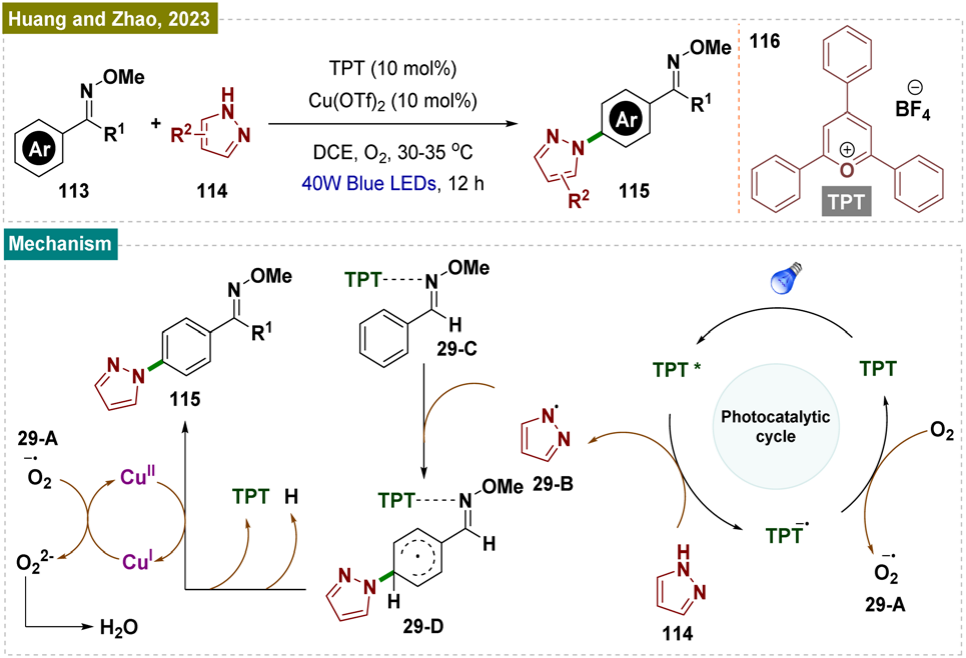
*Para*-selective C–H bond amination of arenes.

Distinct from Nicewicz's report,^148^ in 2023 Huang, Zhao and their team reported a novel method for *para*-selective C–H amination of aryl oximes 113 using transient steric control (Scheme 29).^170^ A preliminary mechanistic investigation identified an interaction between 2,4,6-triphenylpyrylium tetrafluoroborate (TPT) 116 and the oxime 113, which activates the aromatic ring. Simultaneously, TPT 116 creates steric hindrance at the *ortho*- and *meta*-C–H bonds, directing the pyrazole radical to selectively react at the less hindered *para*-position of the phenyl ring. This approach offers a new strategy for achieving *para*-selective C–H functionalization.

Based on these results and prior studies, a plausible reaction mechanism proposed by the authors has been outlined here in Scheme 29. Upon irradiation, the photocatalyst TPT 116 generates an excited state (TPT*) (*E*_1/2_ (TPT*/TPT˙^−^) = +2.55 V *vs.* SCE), which facilitates a single electron transfer, producing a pyrazole radical 29-B (*E*_1/2_ (114/29-B˙) = +2.21 V *vs.* SCE). The pyrazole radical then reacted with intermediate 29-C at the less hindered *para*-position, forming intermediate 29-D. A subsequent single electron transfer to Cu(ii), followed by aromatization, resulted in the formation of the amination product 115. The role of O_2_ was to oxidize back the Cu(i) to Cu(ii) while generating [O_2_]˙^−^29-A*via* SET.

## 5. Conclusions

In this review, we have provided a comprehensive examination of the recent advancements in photocatalytic regioselective C–H bond functionalization of benzene derivatives, with a focus on the strategies and mechanisms that enable high regioselectivity under mild conditions. By systematically analyzing both transition metal and organic photocatalysts, we have highlighted how SET processes and radical intermediates have been harnessed to achieve selective functionalization at the *ortho-*, *meta-*, and *para*-positions of aromatic rings. These methodologies not only enhance the efficiency of C–H activation but also align with the principles of green chemistry, minimizing the need for prefunctionalization and reducing the environmental impact of synthetic processes.

Despite these successes, challenges remain, particularly in enhancing functional group compatibility, reaction scalability, and improving overall efficiency. Future efforts should focus on the design of more sustainable photocatalytic systems that avoid the use of rare metals, while maintaining or even improving selectivity. Moreover, combining photocatalysis with other catalytic strategies, such as HAT and dual-metal catalysis, offers promising avenues for further expanding the reaction scope and addressing current limitations in substrate generality. In this regard, advances in computational modeling and mechanistic understanding will be crucial for guiding the design of next generation photocatalysts. Furthermore, applying these catalytic strategies in the late-stage functionalization of complex, bioactive molecules and in industrial settings will be key to unlocking the full potential of photocatalytic C–H functionalization.

Overall, this review serves as a foundation for the continued development of regioselective photocatalytic C–H functionalization. The combination of detailed mechanistic understanding and innovative catalyst design will undoubtedly lead to new synthetic methods and offer valuable tools for fields such as drug discovery and materials science.

## Data availability

No primary research results have been included and no new data were generated or analyzed as part of this review.

## Author contributions

J. H., S. P. and S. W. developed the concept, wrote the review, and contributed equally. S. D. developed the concept and revised and edited this review.

## Conflicts of interest

There are no conflicts to declare.
